# Brain DNA methylation, *APOE* genotype and Alzheimer’s disease neuropathology in a large postmortem cohort

**DOI:** 10.1038/s43587-026-01243-7

**Published:** 2026-10-12

**Authors:** Guanhong Miao, Cheng Gao, Lei Yu, Jingyun Yang, Yun Li, Francine Grodstein, Julie A. Schneider, Lisa L. Barnes, Philip L. De Jager, Bing Yao, David A. Bennett, Jinying Zhao

**Affiliations:** 1 Health Informatics Institute, University of South Florida, Tampa, FL USA; 2 Rush Alzheimer’s Disease Center & Department of Neurological Sciences, Rush University Medical Center, Chicago, IL USA; 3 Department of Genetics, University of North Carolina at Chapel Hill, Chapel Hill, NC USA; 4 Rush Alzheimer’s Disease Center & Department of Internal Medicine, Rush University Medical Center, Chicago, IL USA; 5 Center for Translational & Computational Neuroimmunology, Department of Neurology and the Taub Institute for Research on Alzheimer’s Disease and the Aging Brain, Columbia University Medical Center, New York, NY USA; 6 Department of Human Genetics, Emory University School of Medicine, Atlanta, GA USA

**Keywords:** Alzheimer's disease, DNA methylation, Ageing

## Abstract

DNA methylation (DNAm) plays a critical role in Alzheimer’s disease (AD). Previous epigenome-wide association studies using 450K arrays captured limited CpG coverage and often focused on a single neuropathological measure. Here we profiled DNAm in 1,250 postmortem prefrontal cortex samples from the Rush Alzheimer’s Disease cohorts using the Infinium MethylationEPIC v2 array (~930,000 CpGs) and performed epigenome-wide association studies of multiple neuropathologic measures, including AD diagnosis, amyloid-β and tau tangles. We identified 107 differentially methylated probes associated with AD neuropathology, 38 of which were replicated in an independent cohort (*n* = 139). DNAm also partially mediated the effect of *APOE* genotype on AD diagnosis (proportion mediated: 8.6%, *P* = 2 × 10^−6^). Moreover, 39 identified genes (for example, *RIN3*, *LGMN*, *LHPP*) were differentially expressed across major brain cell types, including neurons and astrocytes. These findings expand the epigenetic landscape of AD neuropathology and reveal DNAm as a mediator of *APOE*-related genetic risk.

## Abstract (web summary)

A large-scale study identified 107 brain DNA methylation sites linked to Alzheimer’s pathology, with 38 replicated independently. DNA methylation partially mediated the association between *APOE* genotype and Alzheimer’s diagnosis.

## Main

AD is characterized clinically by progressive cognitive decline, and pathologically by amyloid plaques, neurofibrillary tangles and loss of neurons in the brain. A comprehensive understanding of its underlying mechanisms is essential for developing diagnostic and therapeutic tools against AD.

DNA covalent modifications represent key epigenetic mechanisms that regulate numerous cellular and biological processes^1,2^. Among the various known DNA base modifications, methylation at the 5-carbon position of cytosine, termed 5-methylcytosine (5mC), is the most abundant and extensively studied epigenetic mark in the mammalian genome^2^. Growing evidence indicates that 5mC plays a critical role in regulating neurogenesis^3^, memory and learning, and in synaptic plasticity^4^. In human population studies, we^5–8^ and others^9–13^ have reported associations between altered 5mC in postmortem brain tissue and AD-related neuropathology.

In addition, several epigenome-wide association studies (EWAS)^5,9,10^, including meta-analyses integrating brain DNAm data across multiple cohorts and brain regions^11,12^, have identified differential methylation patterns associated with AD neuropathology. For example, CpG sites in several genes, such as *ANK1*, *RHBDF2* and the *HOXA* gene cluster, have shown robust associations with AD pathology (primarily Braak staging) across regions, such as the prefrontal cortex (PFC), temporal gyrus and entorhinal cortex^5,9–12^. Notably, a recent study profiled DNAm using the Illumina EPIC v1 array in two cortical regions (dorsolateral prefrontal cortex (DLPFC) and occipital cortex) from 631 donors in the Brains for Dementia Research (BDR) cohort^10^. By meta-analyzing the data with previous cortical methylation studies, the authors identified epigenetic signatures associated with multiple AD neuropathologic measures, including Braak stage, Thal phase and Consortium to Establish a Registry for Alzheimer’s Disease (CERAD) score. These findings highlight the value of integrating multiple neuropathological measures to uncover robust epigenetic signatures of AD. Nonetheless, most prior EWAS have focused on a single pathological trait (for example, Braak stage) and primarily relied on the Infinium HumanMethylation450 array, which covers a limited portion of the methylome compared to newer platforms.

The *APOE* gene, which encodes apolipoprotein E, is the strongest genetic determinant of late-onset AD. The *APOE* ε4 allele increases AD risk in a dose-dependent manner and accelerates amyloid-β (Aβ) and tau pathology, whereas the ε2 allele is protective. Although these genetic associations are well established, emerging evidence indicates that *APOE*-related risk may also involve epigenetic regulation. Several studies have identified differential DNAm both in the *APOE* locus and at distal genomic regions that are associated with *APOE* genotype and AD^14,15^. For example, methylation changes in the *APOE* promoter and nearby genes (*TOMM40*, *APOE*, *APOC1*) have been linked to altered *APOE* expression, tau pathology and AD-related cerebrospinal fluid biomarkers^14,15^. Additional work has reported *APOE*-related differentially methylated probes (DMPs) across multiple brain regions, suggesting that *APOE* genotype exerts broad epigenetic influence on AD-relevant pathways^15,16^. Collectively, these findings highlight that *APOE* contributes to AD risk not only through its coding variation, but also through epigenetic modifications that modulate gene regulation in the brain. Understanding the interplay between *APOE* genotype, DNAm and AD pathology may thus provide insights into disease mechanisms and identify epigenetic biomarkers or therapeutic targets for precision medicine approaches in AD.

In this study, we investigated the association between DNAm (quantified by the Infinium MethylationEPIC v2 array, which assesses more than 935,000 CpG sites) and multiple measures of AD neuropathologies (neuropathologic diagnosis of AD, Aβ load, paired helical filaments tau (PHFtau) tangles) in 1,250 postmortem brain samples (DLPFC) from the Rush Alzheimer’s Disease Center (RADC) cohorts. These cohorts include five clinical–pathologic studies of aging with detailed phenotypic data^17–20^. Findings from the RADC cohorts were replicated using an independent dataset of 139 DLPFC samples from the Netherlands Brain Bank (NBB)^21^. In addition, we evaluated whether DNAm mediates the relationship between *APOE* genotype and AD pathology, and integrated brain DNAm profiles with single-nucleus RNA sequencing (snRNA-seq) data (DLPFC) generated from a subset of the same individuals.

## Results

### Study design and participant characteristics

**Extended Data Fig. 1:**
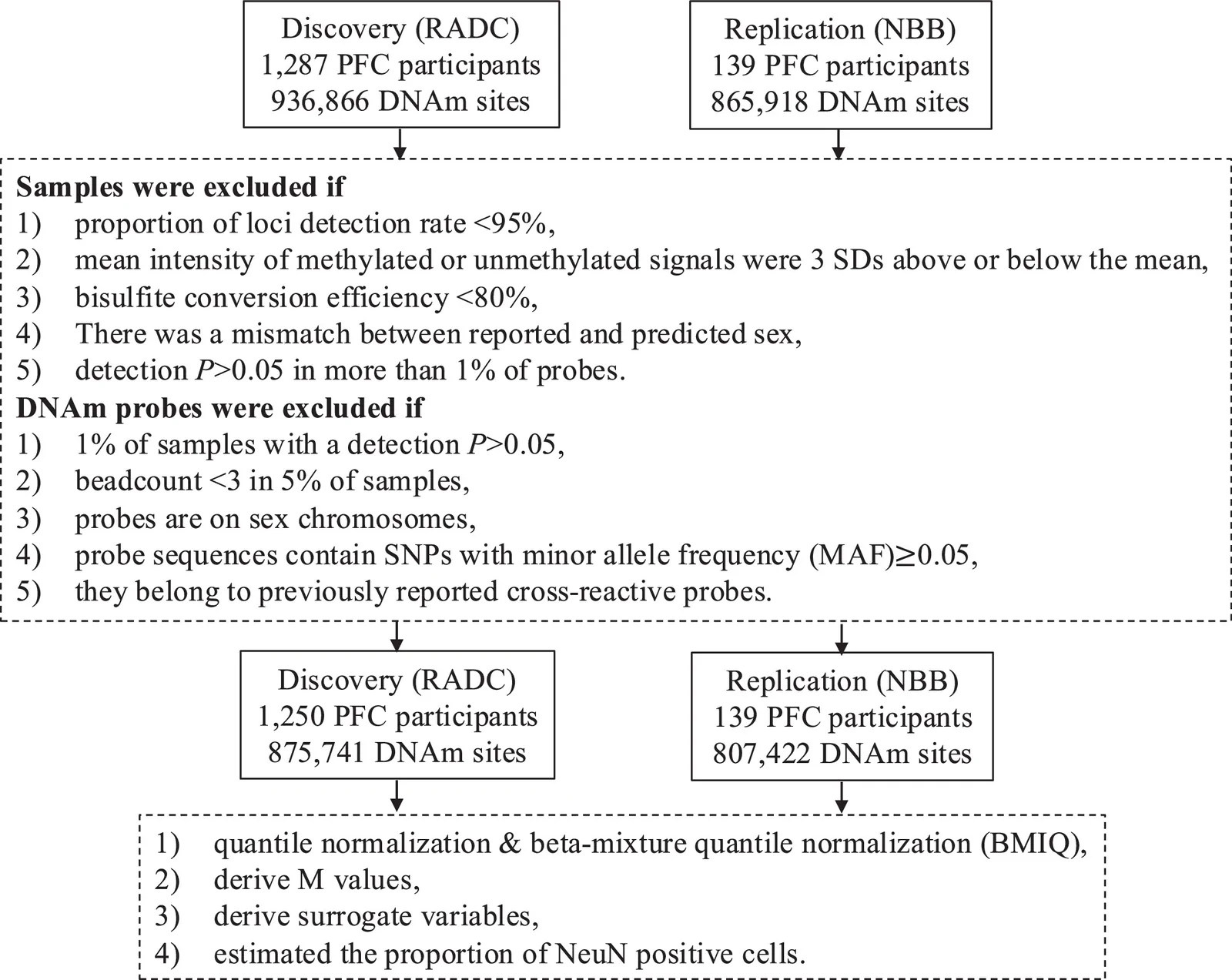
Flowchart illustrating the procedures for sample selection and data preprocessing. RADC: Rush Alzheimer’s Disease Center, NBB: Netherlands Brain Bank, DNAm: DNA methylation, PFC: prefrontal cortex, SD: standard deviation.

Our discovery sample included 1,250 participants from the RADC cohorts^17–20^. The replication sample consisted of 139 participants from the NBB^21^ (Methods). The RADC discovery cohort included 864 (69.1%) women, with a mean (s.d.) age at death of 89.5 (6.7) years (Table 1). As recommended by the National Institute on Aging–Alzheimer’s Association (NIA–AA) guidelines^22^ (Methods), all participants received a neuropathologic diagnosis of no AD, low-likelihood AD, intermediate-likelihood AD or high-likelihood AD based on the Aβ plaque score, Braak NFT score for neurofibrillary pathology, and the CERAD estimate of neuritic plaques. Individuals with an intermediate or high likelihood were classified as having a neuropathologic diagnosis of AD; all others were classified as non-AD^22^. According to the NIA–AA guidelines, 826 (66.1%) RADC participants had a neuropathologic diagnosis of AD (Table 1). The NBB replication cohort included 100 (71.9%) women, with a mean (s.d.) age at death of 80.7 (10.5) years, and 62 (44.6%) had a neuropathologic diagnosis of AD based on clinical evaluation and autopsy confirmation (Methods and Supplementary Table 1). DNAm in DLPFC samples from RADC and NBB was profiled using the Infinium MethylationEPIC v2.0 and v1.0 arrays (Illumina), respectively^23^ (Methods). Quality control and normalization procedures are detailed in the Methods and shown in Extended Data Fig. 1.

**Table 1:** Characteristics of study participants in the discovery sample (RADC, *n* = 1,250)

Characteristics	All (*n* = 1,250)	AD (*n* = 826)	Non-AD (*n* = 424)
Age at death (years)	89.5 (6.7)	90.6 (6.1)	87.4 (7.3)
Female, *n* (%)	864 (69.1)	592 (71.7)	272 (64.2)
Race			
White, *n* (%)	1,166 (93.3)	778 (94.2)	388 (91.5)
African American, *n* (%)	66 (5.3)	39 (4.7)	27 (6.4)
American Indian or Alaska Native, *n* (%)	5 (0.4)	3 (0.4)	2 (0.5)
Other, *n* (%)	13 (1.0)	6 (0.7)	7 (1.6)
Aβ load	1.6 (1.1)	2.2 (0.8)	0.5 (0.7)
Neurofibrillary tangles (PHFtau)	1.7 (1.4)	2.2 (1.5)	0.8 (0.5)
PMI (h)	9.0 (6.8)	8.8 (6.3)	9.5 (7.7)
*APOE* ε4 carrier^a^, *n* (%)	277 (22.7)	229 (29.3)	48 (11.8)

Continuous variables are expressed as mean (s.d.) and qualitative variables as *n* (%). AD, neuropathologic diagnosis of Alzheimer’s disease.

^a^*APOE* ε4 carrier includes the ε4/ε4 carrier and ε3/ε4 carrier. To avoid the potential confounding effects of the ε2 allele, a total of 28 participants with *APOE* ε2/ε4 carrier were excluded from the *APOE* mediation analysis.

### DNAm alterations are associated with AD neuropathology

**Fig. 1:**
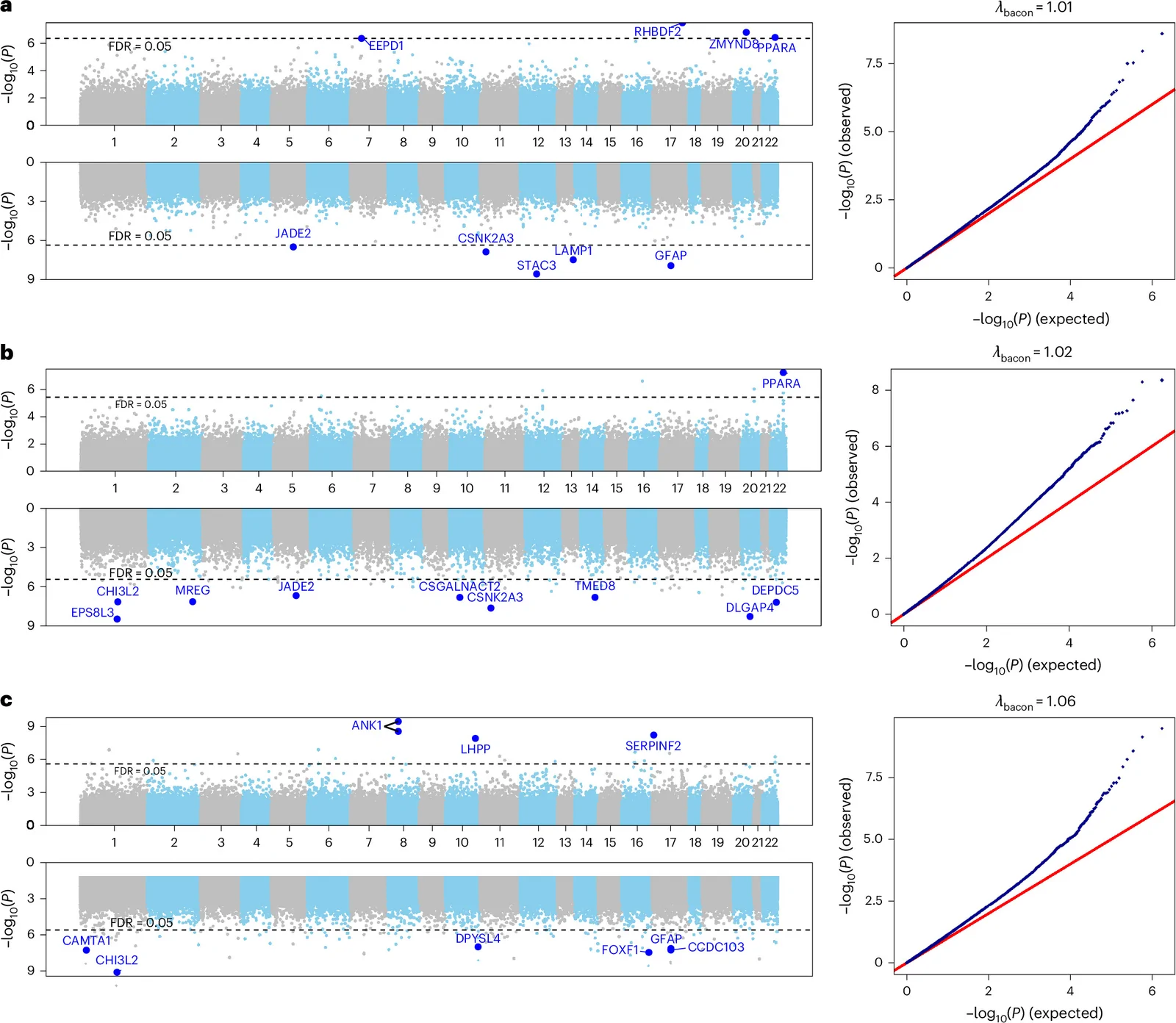
DMPs associated with AD neuropathologies in the RADC cohort (*n* = 1,250). **a**–**c**, Miami plots showing associations between brain DNAm and pathologic AD diagnosis (**a**), Aβ load (**b**) and PHFtau tangle burden (**c**). *P* values were derived from two-sided linear regression, with multiple testing correction performed using the Benjamini–Hochberg FDR method (dashed line, FDR = 0.05). The *x* axis denotes genomic positions across chr. 1–22, and the *y* axis represents −log_10_(*P*) values. Up to ten top genes annotated to the most significant DMPs are labeled for each phenotype. For each phenotype, a corresponding quantile–quantile (QQ) plot shows the expected versus observed –log_10_(*P*) values. Red line represents the expected distribution of −log_10_(*P*) values under the null hypothesis, and *λ*_bacon_ denotes the genomic inflation factor after Bacon correction. Source data

**Extended Data Fig. 2:**
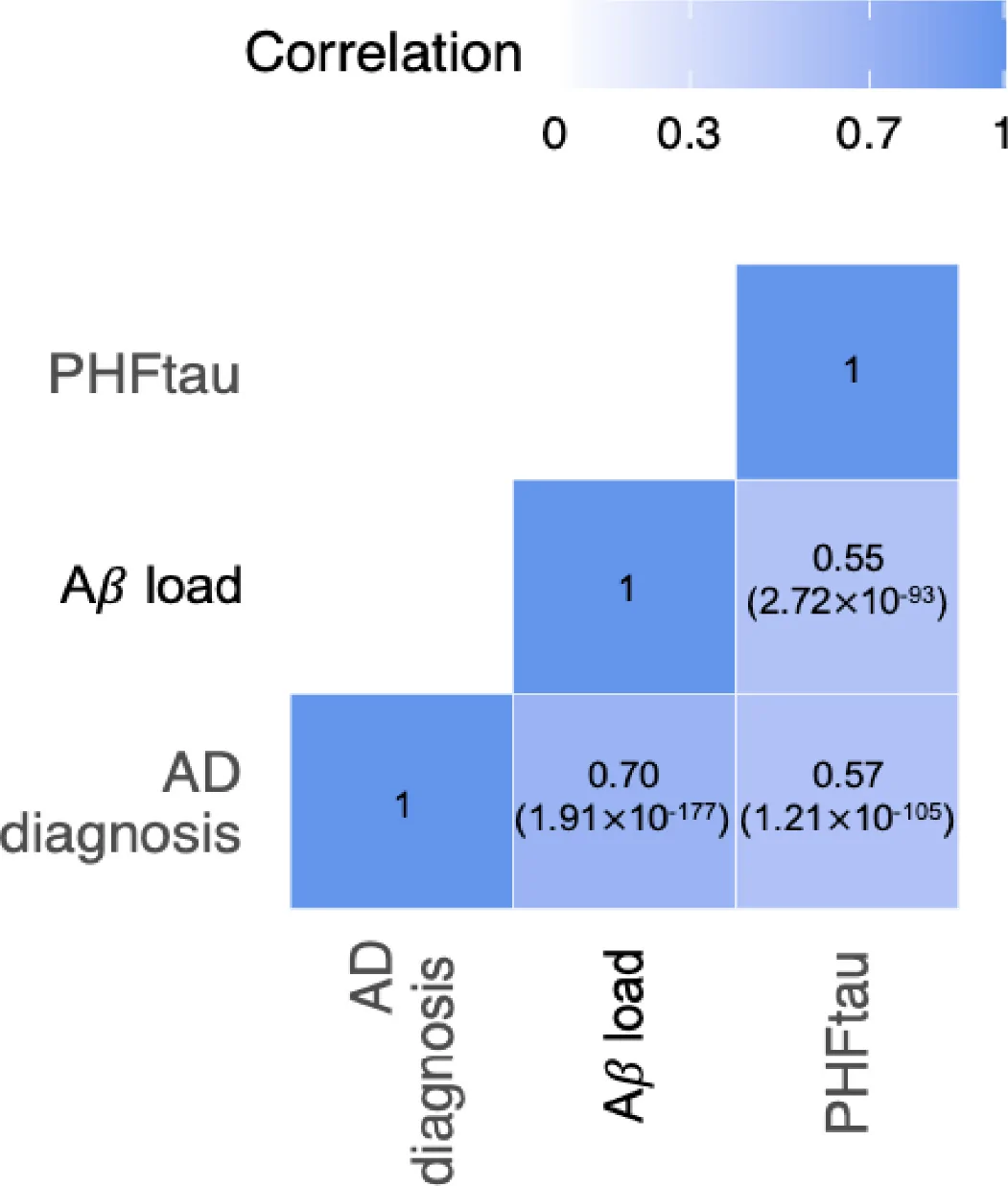
Heatmap showing correlations among AD diagnosis, Aβ load, and PHFtau tangles in the RADC discovery sample (n = 1,250). Each cell displays the Spearman correlation coefficient and corresponding *P* value between the two measures.

**Extended Data Fig. 3:**
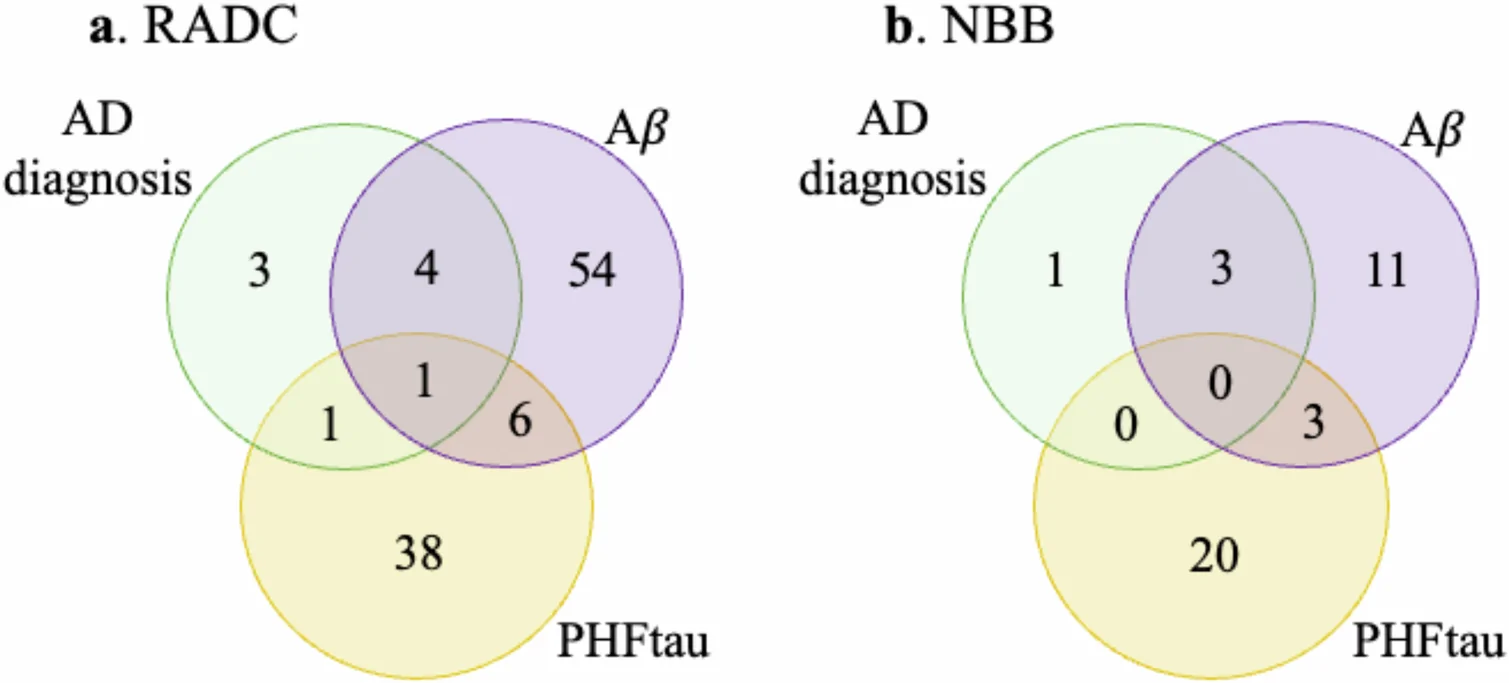
Venn diagrams depicting the number of differentially methylated probes (DMPs) associated with brain neuropathologies. (**a**) Discovery sample (RADC, n = 1,250). (**b**) Replication sample (NBB, n = 139).

Using brain DNAm data from 1,250 RADC discovery samples (DLPFC) profiled by the Infinium MethylationEPIC v2 array, we conducted an EWAS to identify DMPs associated with three neuropathologic measures: (1) neuropathologic diagnosis of AD, (2) Aβ load and (3) PHFtau tangles (Methods and Extended Data Fig. 2). Models were adjusted for age at death, sex, race, postmortem interval (PMI), NeuN-positive cell proportion, experiment plate and unwanted variation captured by the first three surrogate variables from the R package SVA^24^. At a Benjamini–Hochberg false discovery rate (FDR) <0.05, we identified 107 DMPs, 9 (annotated to 17 genes) of which were associated with pathologic AD diagnosis, 65 (annotated to 119 genes) with Aβ load and 46 (annotated to 82 genes) with PHFtau tangles (Fig. 1, Table 2 and Supplementary Tables 2 and 3). Among these DMPs, 12 (for example, cg08550059, cg05959720, cg20668546) were associated with 2 or more AD pathologies, and 1 probe (cg08550059) was associated with all 3 (Extended Data Fig. 3).

**Table 2:** DMPs associated with neuropathologic diagnosis of AD

DMP	Chromosome	Position (bp)	GREAT annotation (distance to site)	Discovery (RADC, *n* = 1,250)			Replication (NBB, *n* = 139)^a^			DMP identified in previous EWAS with same direction?	In 450K array?
				Coefficient (95% CI)	*P*	FDR	Coefficient (95% CI)	*P*	FDR		
cg08550059	5	134509443	*CDKN2AIPNL* (−97546), *JADE2* (−16214)	−0.05 (−0.06, −0.03)	3.18 × 10^−7^	0.040				No	No
**cg00744233**	**7**	**36231662**	***ANLN*** **(−158144),** ***EEPD1*** **(**+**78514)**	**0.10 (0.06, 0.14)**	**4.39** × **10**^−**7**^	**0.043**	**0.15 (0.05, 0.26)**	**0.005**	**0.007**	No	No
**cg05959720**	**11**	**10899263**	***ZBED5*** **(−41191),** ***CSNK2A3*** (**+454094)**	**−0.07 (−0.10, −0.05)**	**1.34** × **10**^−**7**^	**0.023**	**−0.14 (−0.21, −0.06)**	**0.001**	**0.002**	No	No
cg01643513	12	57280295	*STAC3* (−29103)	−0.07 (−0.09, −0.05)	2.68 × 10^−9^	0.002	−0.04 (−0.11, 0.04)	0.310	0.310	No	No
**cg04923178**	**13**	**113274396**	***LAMP1*** **(−22845),** ***CUL4A*** (**+64853)**	**−0.07 (−0.09, −0.04)**	**3.31** × **10**^−**8**^	**0.007**	**−0.12 (−0.19, −0.04)**	**0.003**	**0.005**	No	No
cg20668546	17	44911955	*GFAP* (+3531), *CCDC103* (+12182)	−0.15 (−0.20, −0.10)	1.17 × 10^−8^	0.005	−0.12 (−0.25, 0.01)	0.073	0.088	Yes	Yes
cg22975017	17	76495725	*RHBDF2* (+5682), *AANAT* (+42375)	0.09 (0.06, 0.13)	3.14 × 10^−8^	0.007				No	No
**cg00506062**	**20**	**47352421**	***ZMYND8*** **(+4276),** ***EYA2*** **(+457798)**	**0.08 (0.05, 0.10)**	**1.58** × **10**^−**7**^	**0.023**	**0.29 (0.15, 0.44)**	**8.02** × **10**^−**5**^	**4.80** × **10**^−**4**^	No	No
cg26467296	22	46073492	*WNT7B* (−96364), *PPARA* (−78392)	0.06 (0.04, 0.09)	3.73 × 10^−7^	0.041				No	No

DMP information was provided corresponding to chromosomal location (GRCh38 genomic annotation) and nearby genes with a TSS upstream (−) or downstream (+) (from GREAT annotation). The regression coefficient was obtained from linear regression, adjusting for age at death, sex, race (white or nonwhite), PMI, proportion of NeuN-positive cells, experiment plate and the first three surrogate variables. ^a^Among nine DMPs identified in the RADC discovery sample, six were also available in the NBB replication sample. Multiple testing in RADC adjusted for 875,741 hypotheses, while multiple testing in NBB adjusted for 6 association hypotheses that were significant in RADC and available in NBB. DMPs replicated in the NBB replication sample are shown in bold. All statistical tests were two-sided.

**Extended Data Fig. 4:**
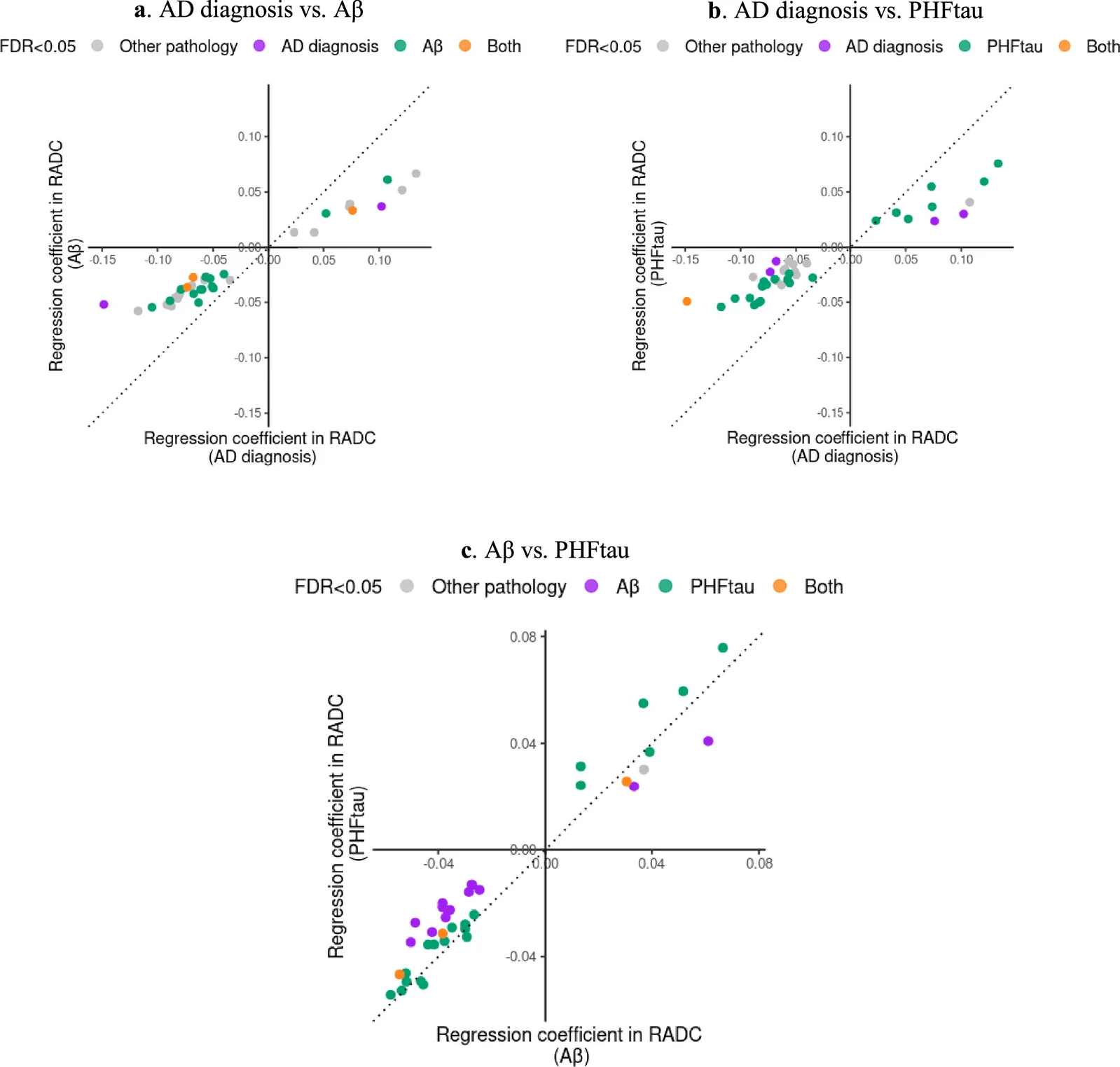
Comparison of the effect sizes (regression coefficients) and association direction of 38 DMPs with AD neuropathologies (n = 1,250). The 38 DMPs shown were identified in the RADC discovery cohort and replicated in the NBB cohort. Association direction and effect size were compared across three AD neuropathologies — neuropathologic diagnosis of AD, Aβ load, and PHFtau tangles — with 100% concordance in direction across all three phenotypes (**a**, AD diagnosis versus Aβ load. **b**, AD diagnosis versus PHFtau tangles. **c**, Aβ load versus PHFtau tangles). Each dot represents a single CpG site. Source data

**Extended Data Fig. 5:**
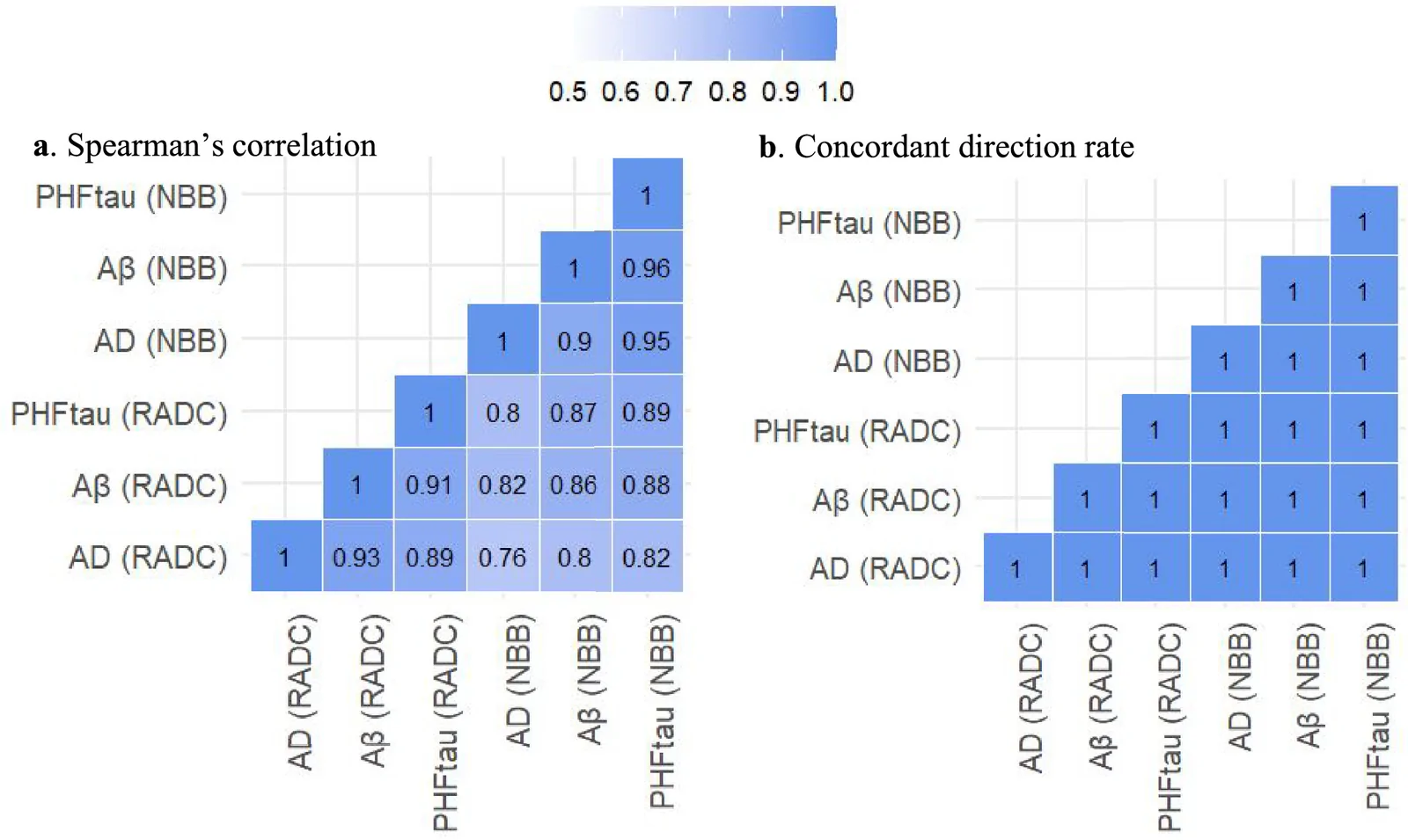
Associations of 38 DMPs replicated in both RADC (discovery, n = 1,250) and NBB (replication, n = 139) cohorts across AD neuropathologies. Regression coefficients in RADC and NBB were calculated using linear models. Spearman correlations (**a**) and concordant direction rates (**b**) of regression coefficients between cohorts (for example, DMP–Aβ in RADC vs. NBB) are shown. ‘AD’ denotes neuropathologic AD diagnosis. Concordant direction rate was calculated as the fraction of the 38 DMPs with consistent directions of association across cohorts.

We next sought to replicate our findings in an independent cohort of 139 NBB participants, in whom AD pathology was characterized by pathologic diagnosis, Aβ stage and Braak stage (Methods). Most DMPs identified in the RADC discovery cohort were also measured and passed quality control in NBB (67% of AD diagnosis-associated DMPs, 77% of Aβ-associated DMPs and 76% of PHFtau-associated DMPs). After multiple testing correction, 38 DMPs (annotated to 67 unique genes) remained significant in the replication analysis, including 4 associated with pathologic AD diagnosis, 17 with Aβ and 23 with Braak stage—all exhibiting consistent directions of effect (Table 2, Extended Data Fig. 3 and Supplementary Tables 2 and 3). Regression coefficients for these 38 unique DMPs were highly correlated across the three neuropathologic traits, with a concordant direction of association for all 38 DMPs in both the discovery and replication cohorts (Extended Data Figs. 4 and 5 and Supplementary Table 4). Moreover, 36 of the 38 DMPs (94.7%) were nominally associated with all three AD pathologic phenotypes (*P* < 0.05) (Supplementary Table 4), although some did not remain significant after correction for multiple testing (Extended Data Fig. 4). Notably, 16 (42%) of the 38 DMPs have also been reported in prior large-scale EWAS of AD in the PFC^10–12^ (Supplementary Table 5).

**Fig. 2:**
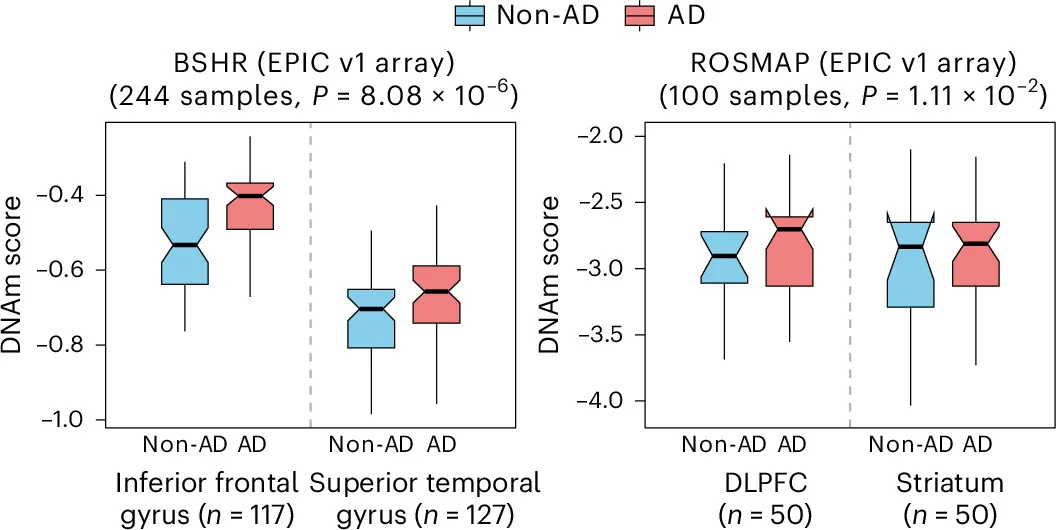
Replication in two independent cohorts. A DNAm score, defined as the weighted sum of 38 AD-associated DMPs confirmed in both RADC and NBB, was significantly associated with a neuropathologic diagnosis of AD in two independent brain samples: BSHR (inferior frontal gyrus, *n* = 117; superior temporal gyrus, *n* = 127) and ROSMAP (DLPFC, *n* = 50; striatum, *n* = 50). All analyses were adjusted for demographic, cellular, technical and regional covariates; ROSMAP analyses were additionally adjusted for race. *P* values were derived from two-sided linear mixed-effects models, with participant included as a random effect to account for the correlation between DNAm measurements from the two brain regions contributed by each individual. In the boxplots, the central box represents the lower, median and upper quartiles, and the whiskers extend to the most extreme data points within 1.5× the interquartile range. Source data

To further validate our findings, we constructed a DNAm score based on these 38 DMPs (Methods) and applied it to two independent, cross-region brain datasets profiled with the Infinium MethylationEPIC v1 array: Banner Sun Health Research Institute^25^ (BSHR; 117 inferior frontal gyrus samples, 127 superior temporal gyrus samples) and ROSMAP^26^ (independent RADC subset; 50 PFC samples, 50 striatum samples) (Methods and Supplementary Table 1). After covariate and batch adjustment, the methylation score was significantly higher in individuals with pathologic AD than those without AD in both the BSHR and ROSMAP cohorts (Fig. 2).

To identify genomic regions containing multiple DMPs, we performed differentially methylated region (DMR) analysis for each neuropathology measure using the software comb-p^27^ (Methods). This analysis identified 1 DMR associated with AD diagnosis, 13 associated with Aβ and 20 associated with PHFtau (Supplementary Table 6). Together, these DMRs encompassed 102 unique CpG sites, 47 of which overlapped with DMPs identified in our study or prior EWAS. These findings highlight key genomic regions of coordinated methylation changes that may contribute to AD pathogenesis.

### DNAm mediates the association between *APOE* genotype and neuropathologic diagnosis of AD

To test whether DNAm mediates the effect of *APOE* genotype on AD diagnosis, we performed mediation analysis using the R package mediation^28^ (Methods). Among the 38 DMPs identified in RADC and replicated in NBB cohorts, 23 (60.5%) were associated with *APOE* ε4 carrier status (yes or no) at the nominal significance threshold of *P* < 0.05 (Supplementary Table 7). Further adjustment for *APOE* genotype slightly attenuated the associations between these 23 DMPs and neuropathologic AD diagnosis (Supplementary Table 7).

**Fig. 3:**
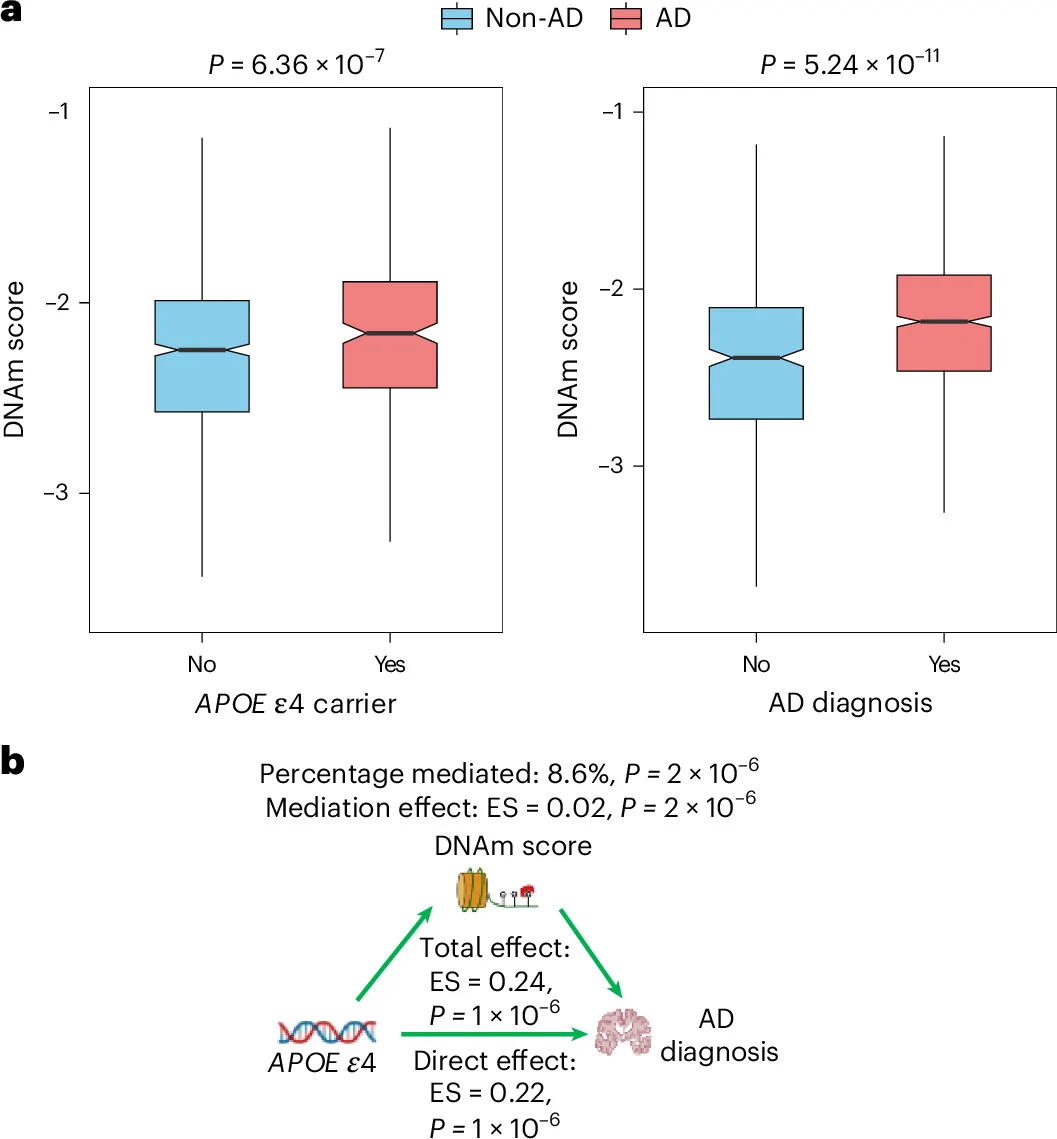
DNAm partially mediates the effect of *APOE* genotype on neuropathologic AD. A total of 1,183 RADC participants with available *APOE* genotype data were included in this analysis. **a**, DNAm score constructed from 23 *APOE*-associated DMPs was significantly associated with both *APOE* ε4 carrier status (linear regression) and AD diagnosis (logistic regression). In the boxplots, the central box shows the lower, median and upper quartiles, and the whiskers extend to the most extreme data points within 1.5× the interquartile range. **b**, DNAm score partially mediated the association between *APOE* genotype and AD diagnosis (95% CI 4.3–14.6%, *P* = 2 × 10^−^^6^). Total, direct and mediation effects were estimated using mediation analysis with the R package mediation. All models were adjusted for demographic, cellular and technical covariates, and all statistical tests were two-sided. ES, effect size. Source data

Of the 23 *APOE*-associated DMPs, 20 partially mediated the effect of *APOE* genotype on AD diagnosis, with proportions mediated ranging from 1.8% to 5.9% after covariate adjustment and multiple testing correction (Supplementary Table 7). Furthermore, we constructed an *APOE*-associated DNAm score based on these 23 DMPs (Methods). This score was significantly associated with both *APOE* genotype and pathologic AD diagnosis (Fig. 3a) and mediated 8.6% of the *APOE*–AD association (95% confidence interval (CI) 4.3–14.6%, *P* = 2 × 10^−^^6^) (Fig. 3b).

### Cell-type-specific expression of genes associated with AD neuropathology

**Extended Data Fig. 6:**
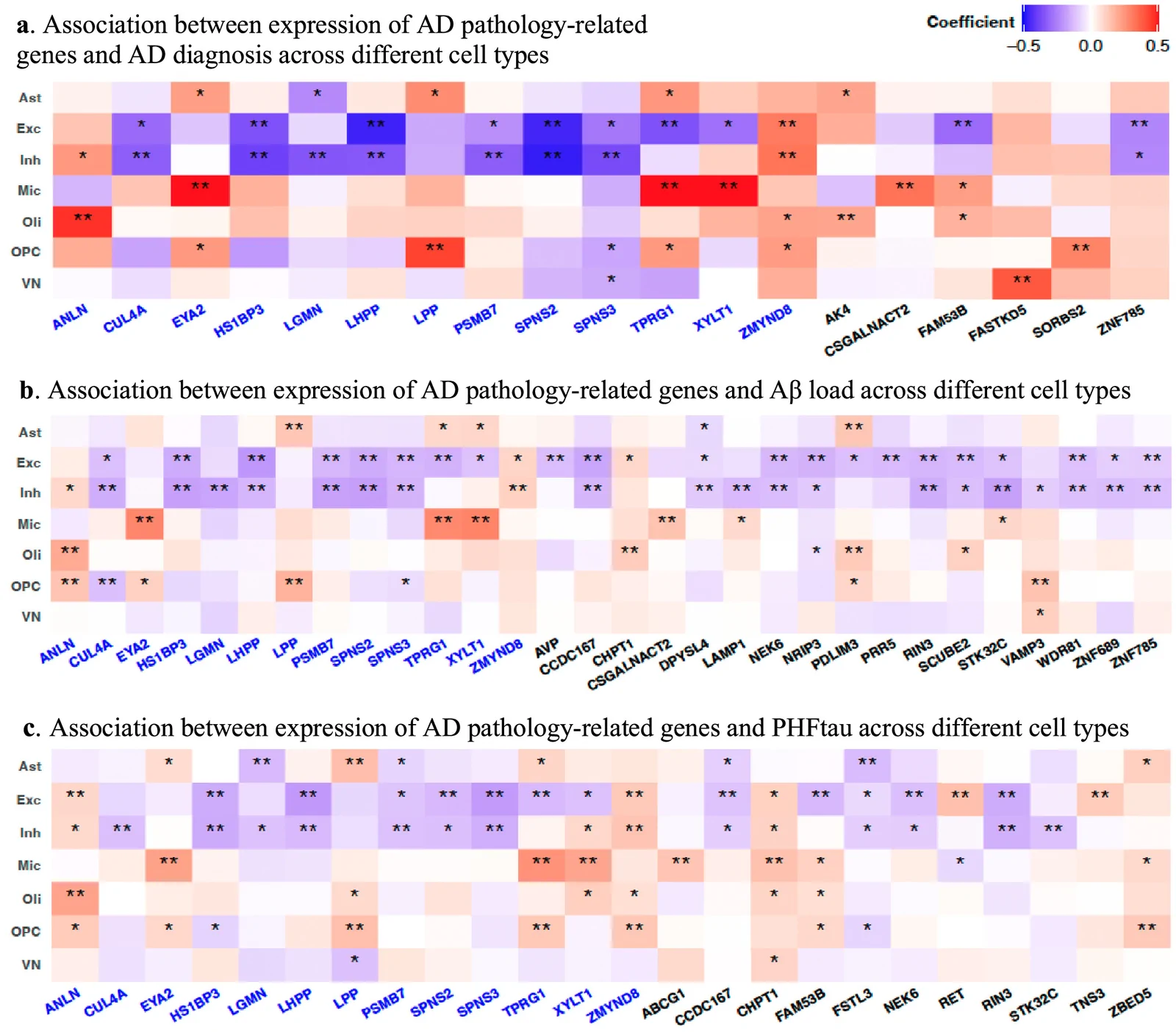
Cell-type–specific expression of candidate genes associated with AD neuropathology in the RADC discovery cohort. Of 67 EWAS-identified genes (differentially methylated in bulk brain tissue of RADC samples and replicated in an independent sample from NBB), 39 (58.2%) were also differentially expressed across major brain cell types (FDR < 0.05) based on snRNA-seq data from 437 RADC brain samples. Thirteen of these genes (highlighted in blue) were significantly associated with all three neuropathologic measures (**a**: AD diagnosis, **b**: Aβ load, **c**: PHFtau tangles). Color intensity reflects regression coefficients from linear models adjusting for age at death, sex, postmortem interval, and batch effects. Each subfigure displays genes linked to the specific pathology (FDR < 0.05) in ≥1 cell type. Cell types: Ast, astrocytes; Exc, excitatory neurons; Inh, inhibitory neurons; Mic, microglia; Oli, oligodendrocytes; OPC, oligodendrocyte precursors; VN, vascular niche. **FDR < 0.05, *P < 0.05. Source data

Using snRNA-seq data from 437 DLPFC samples in RADC (Methods), we examined whether genes harboring DMPs identified in bulk DLPFC were also differentially expressed across major brain cell types. Of the 67 genes harboring 38 DMPs identified in RADC and replicated in NBB cohorts, 39 (for example, *RIN3*, *LGMN*, *LPP*) showed differential expression across cell types (Extended Data Fig. 6). Notably, the expression levels of 13 genes (for example, *LGMN*, *LHPP*, *SPNS2*) were also significantly associated with all three neuropathologic traits (FDR < 0.05). Compared to individuals without AD, most of these genes were downregulated in excitatory and inhibitory neurons, but upregulated in microglia, oligodendrocyte precursor cells and oligodendrocytes in AD cases. These findings highlight cell-type-specific transcriptional changes that mirror DNAm differences, suggesting coordinated epigenetic and transcriptional regulation in AD neuropathology.

### Genetic coregulation and enrichment analysis

To assess whether DNAm and gene expression share common genetic regulation, we conducted colocalization analysis using the R package coloc^29^ (Methods). At *PP*_4_ > 0.8 (that is, a high posterior probability that a single variant regulates both DNAm and gene expression), DNAm and expression of *RIN3* were genetically coregulated in microglia (Supplementary Table 8).

**Extended Data Fig. 7:**
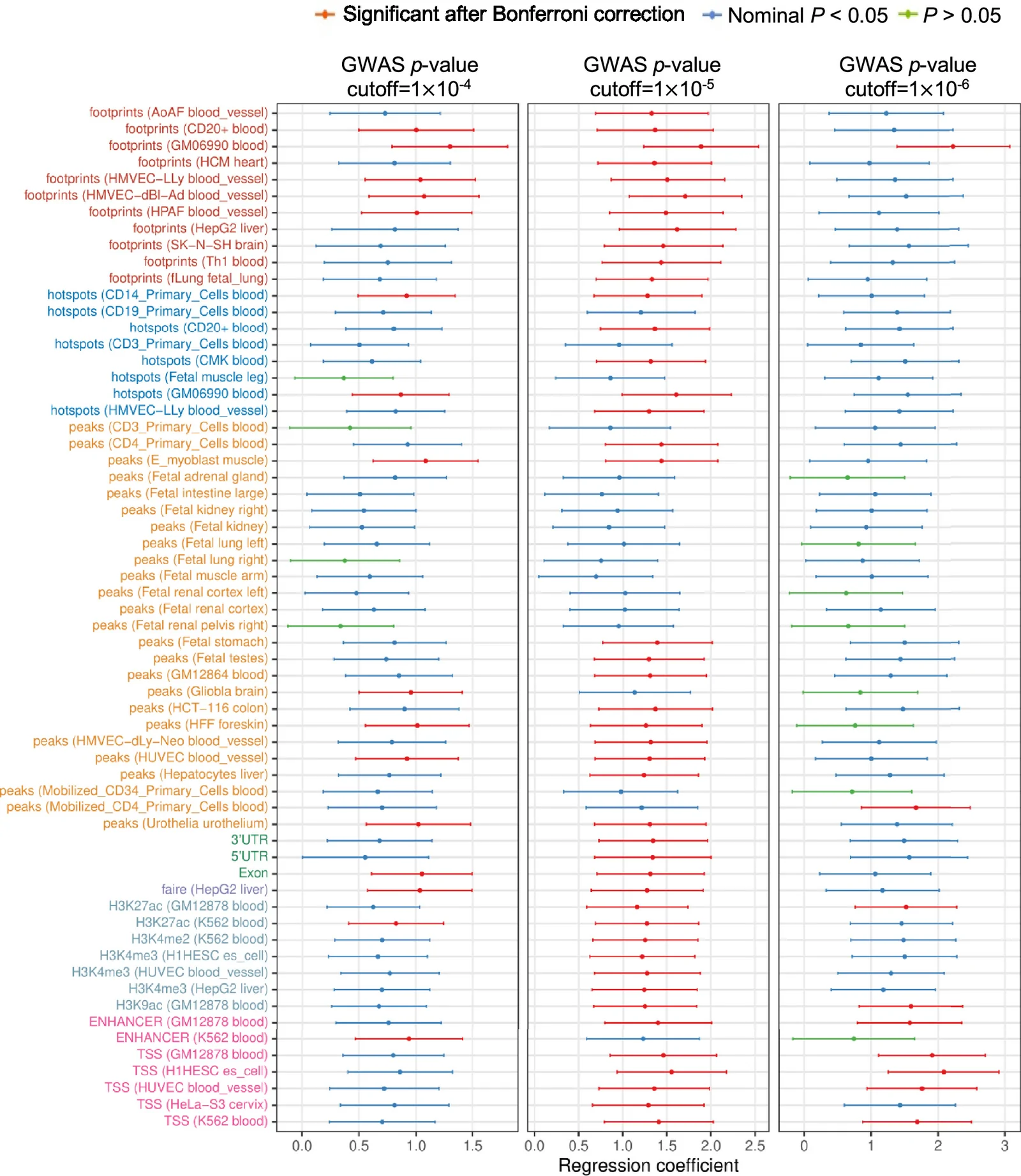
Cell-type enrichment of genomic features identified by GARFIELD across multiple GWAS *P* value thresholds. Colors indicate annotation categories (footprints, hotspots, peaks, genic features, FAIRE, histone modifications, chromatin states) in different tissues and cell types, and error bars show 95% confidence intervals. X-axis: regression coefficients (log odds ratio). TSS: transcription start site. Source data

To assess functional relevance, we examined whether the identified DMPs were enriched in tissue- and cell-type-specific genomic elements using GARFIELD v.2.0^30^ (Methods). After correction for multiple testing, significant enrichments were observed across multiple genome-wide association studies (GWAS) significance thresholds (Extended Data Fig. 7 and Supplementary Table 9), predominantly in active regulatory regions, including accessible chromatin (peaks, hotspots and footprints), transcription start site (TSS) and enhancer chromatin states, as well as activation-associated histone marks (H3K4me3, H3K27ac and H3K9ac), indicating that these DMPs preferentially localize to regions with active transcriptional regulatory function.

### Sensitivity analysis for population stratification adjustment

**Extended Data Fig. 8:**
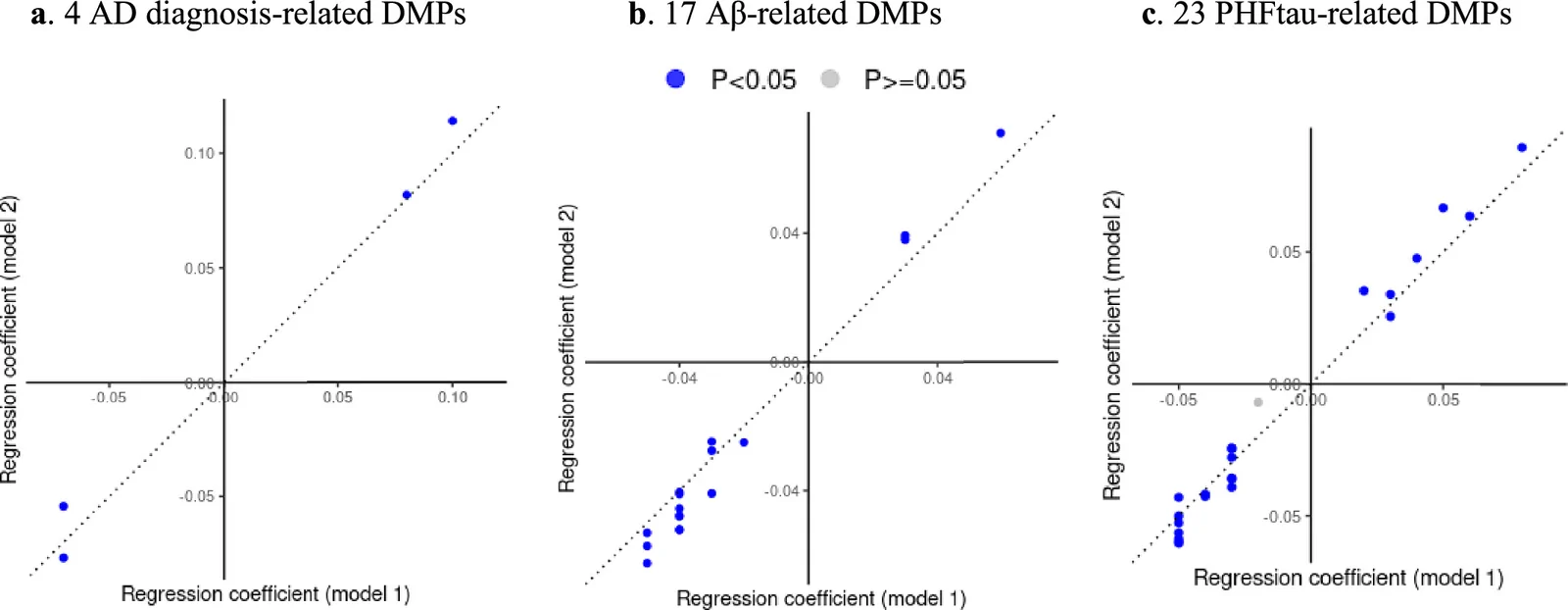
Concordance of DMP effect sizes between models adjusting for binary race (whites vs. nonwhites) and adjusting for the first five genetic PCs. Scatterplots compare regression coefficients from the primary model adjusting for self-reported race as a binary variable (white vs. nonwhite; Model 1, x-axis; n = 1,250) against those from a sensitivity model adjusting for the first five genetic principal components (PCs; Model 2, y-axis; n = 927). Each point represents a replicated DMP reaching significance in both discovery and replication cohorts under Model 1. The dotted line denotes the line of identity (y = x). Points are colored by significance in Model 2: blue indicates P < 0.05 and grey indicates P ≥ 0.05. Across all three phenotypes (**a**: AD diagnosis, **b**: Aβ load, **c**: PHFtau tangles), effect sizes were highly concordant between the two models, with 100% agreement in the direction of association, demonstrating that our primary findings are robust to the choice of ancestry adjustment. Source data

To confirm that adjusting for self-reported race as a binary covariate (white (93.3%) versus nonwhite (6.7%)) adequately controlled for population stratification, we conducted a sensitivity analysis in the 927 RADC participants (74.2% of the full analytic sample) with available genetic data, replacing the binary race covariate with the top five genetic principal components (PCs). Regression coefficients for all AD pathology-associated DMPs were highly concordant between the two adjustment strategies across all three neuropathological phenotypes (Extended Data Fig. 8 and Supplementary Table 10), with fully concordant effect directions and nearly all DMPs remaining nominally significant (*P* < 0.05) under both approaches. These results confirm that the binary race covariate provides adequate control for population stratification in this predominantly white cohort.

## Discussion

In this large-scale EWAS of brain DNAm across multiple measures of AD neuropathologies, including neuropathologic AD diagnosis, Aβ load and neurofibrillary tangles, we identified 107 DMPs, 12 of which were associated with two or more pathologies. Of these, 38 DMPs (~36%) were replicated in an independent cohort of 139 DLPFC samples, including 6 associated with two pathologies and 32 associated with a single pathology. In two additional brain datasets, a DNAm score derived from these 38 DMPs was significantly higher in individuals with a pathologic AD diagnosis than in those without, suggesting the robustness of our findings. We further demonstrated that DNAm partially mediates the effect of *APOE* genotype on AD diagnosis, highlighting a potential epigenetic mechanism underlying genetic risk. Integrative analyses with snRNA-seq data from the same individuals revealed differential expression of several genes (for example, *RIN3*, *LGMN*, *LPP*) across major brain cell types. Together, these results provide additional evidence that altered DNAm contributes to AD neuropathology and suggest coordinated epigenetic and transcriptional regulation across different brain cell types.

Several findings merit discussion. First, of the 38 DMPs (annotated to 67 genes) replicated across cohorts, 16 (for example, *ANK1*, *CAMTA1*, *RHOB*, *LPP*) have been reported in prior EWAS of AD in the same PFC region^10–12^ (Supplementary Table 4). Four genes overlap with AD GWAS loci (*RIN3*, *WDR81*, *TMEM121*, *FOXF1*)^31,32^, and seven (*GFAP*, *DPYS*, *AK4*, *DNAJC6*, *RET*, *RIN3*, *LPP*) were nominated as potential drug targets for AD in the Agora database^33^. In addition, 39 AD pathology-associated genes (for example, *CUL4A*, *EYA2*, *PSMB7*, *XYLT1*, *ZMYND8*) showed differential expression across major brain cell types (Extended Data Fig. 6), suggesting their potential functional relevance. Second, DNAm alterations identified in our EWAS were consistently associated with three neuropathologic traits (pathologic AD diagnosis, Aβ load, neurofibrillary tangles). Notably, methylation changes in *JADE2*, *CHI3L2* and *CSNK2A3* were associated with all three traits in the RADC discovery cohort. *JADE2* has been shown to modulate *APOE* ε2 allele on AD risk^34^ and regulate microglial inflammation, neurogenesis and tau-related neurodegeneration via *LSD1*^34^. *CHI3L2* is implicated in immune and inflammatory responses, similar to *CHI3L1*, a pro-inflammatory factor in neurodegeneration^35^. *CSNK2A3*, a subunit of casein kinase 2, is an emerging target in neurological and autoimmune disorders^36^, although its role in AD remains unclear. Collectively, these findings support the role of DNAm in immune dysregulation and neuroinflammation, key drivers of AD pathology. Third, snRNA-seq revealed that 39 of the 67 genes annotated to replicated DMPs were also differentially expressed. Most were downregulated in neurons but upregulated in non-neuronal cells (microglia, oligodendrocyte precursor cells, oligodendrocytes) in AD cases, highlighting cell-type-specific transcriptional heterogeneity associated with methylation changes. Fourth, a methylation score derived from the 38 replicated DMPs distinguished individuals with pathologic AD from those without in two independent brain datasets, supporting the robustness of these candidate genes across populations and brain regions. Finally, mediation analysis revealed that the identified DMPs accounted for 8.6% of the effect of *APOE* genotype on AD diagnosis, indicating that epigenetic alterations partially mediate genetic risk. Notably, none of these DMPs were located on chr. 19, where *APOE* resides, suggesting that *APOE*-driven biology, such as lipid metabolism, immune–microglial activation or vascular processes^37^, may remodel DNAm at distal, functionally related loci. Potential mechanisms underlying these *trans*-acting epigenetic effects may include methylation-mediated regulation of genes involved in *APOE*-related pathways (for example, lipid metabolism, neuroinflammation, amyloid processing), interactions between *APOE* and other AD-associated genes, and contributions from systemic inflammation or environmental influences^38–40^.

Our results revealed that some CpG sites were associated with more than one pathological trait, suggesting shared molecular mechanisms underlying multiple aspects of AD pathology. Across all 38 replicated DMPs, the direction of effect was fully concordant across all three neuropathological phenotypes (AD diagnosis, Aβ load and PHFtau tangles). Furthermore, 36 of 38 DMPs (94.7%) reached nominal significance (*P* < 0.05) in each phenotype, suggesting that these CpGs reflect epigenetic changes that are not specific to a single pathological trait but are instead broadly shared across AD neuropathology. After correction for multiple testing, several replicated CpGs (cg10045881, cg16215084, cg25917732) were associated with both Aβ and tau pathology, consistent with prior evidence that these lesions frequently co-occur and may arise from overlapping biological processes, such as inflammation, immune activation and cellular stress^38,41^. By contrast, other CpGs were associated with only one trait after multiple testing, indicating trait-specific epigenetic alterations. Specifically, 20 of the 38 replicated CpGs were only associated with tau pathology at FDR < 0.05, potentially reflecting tau-specific neurodegenerative mechanisms, such as intracellular microtubule dysfunction and axonal transport^41^. Similarly, 14 CpGs were only associated with Aβ load at FDR < 0.05, likely reflecting amyloid-related processes such as APP processing, microglial-mediated clearance and cerebral amyloid angiopathy^38,41^. Taken together, the partial overlap among AD pathology-associated DMPs indicates that both shared and distinct epigenetic mechanisms contribute to the individual measure of AD neuropathology. These findings highlight the complex, multifactorial nature of AD and underscore the value of integrating multiple complementary phenotypes to achieve a more comprehensive understanding of its molecular underpinnings.

**Extended Data Fig. 9:**
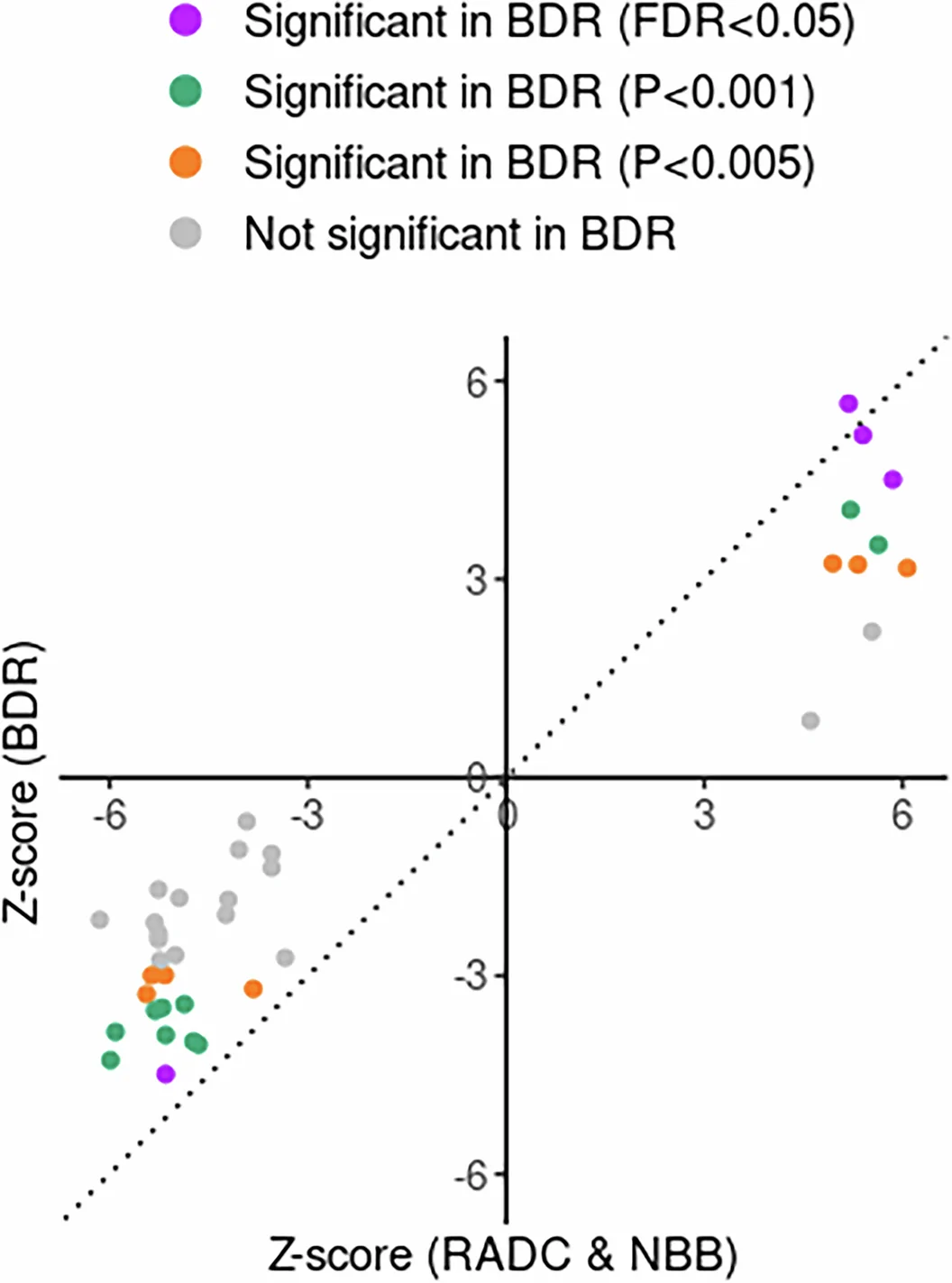
Cross-cohort replication of EWAS results. 21 of 38 DMPs identified in RADC PFC samples (n = 1,250) were also associated with tau pathology in the BDR cohort (n = 610, P < 0.005), showing largely consistent directions of effect. Z-scores = regression coefficient / standard error; each dot = CpG. BDR: Brains for Dementia Research; RADC: Rush Alzheimer’s Disease Center. Z-scores on the x- and y-axes represent the regression coefficient divided by its standard error. Each dot corresponds to a single CpG site. Source data

Our study has several limitations. First, our DNAm data were generated using bulk PFC tissue. Because DNAm is often cell-type- and region-specific, and bulk tissue is a mixture of different cell types, this approach may obscure specific cellular signals. Nevertheless, our findings show substantial overlap with a recent AD EWAS, which used cell-type adjustment based on isolated nuclei from the DLPFC in the BDR cohort^10^. Specifically, more than half of the DMPs identified in our study were also associated with AD pathology in an independent cohort (the BDR cohort), although some did not remain significant after multiple testing (Extended Data Fig. 9). Discrepancies may stem from differences in pathological measurements, participant characteristics and covariates (for example, cell-type proportion, PMI). Future studies should incorporate single-cell technologies across multiple brain regions to move beyond the aggregate signals captured in bulk tissue, enabling the dissection of cell-type- and region-specific epigenetic contributions to AD neuropathology^42^. Second, our study participants primarily consist of highly educated older individuals of European ancestry. Additional studies in diverse populations are needed to increase the generalizability of our findings. Third, although our discovery sample is relatively large (*n* = 1,250), the replication sample size is smaller (*n* = 139), which limits our ability to detect additional probes in the replication phase. Fourth, because of the lack of *APOE* genotype information in other cohorts, we were unable to validate the mediating effect of DNAm on the *APOE*–AD association. The true proportion of the mediating effect is likely larger when multiple brain regions are considered. Future cross-region DNAm profiling will be essential to provide further evidence supporting this mediating role. Fifth, our enrichment analysis utilized resources not exclusively derived from brain tissues. Because different tissues exhibit unique DNAm signatures^43^, these findings require further validation specifically in brain tissues. Finally, like many postmortem studies, our findings are cross-sectional, and it remains unclear whether the observed DNAm differences are a cause or consequence of neuropathologic changes.

In summary, in a large cohort of older individuals with postmortem brain tissue, we identified differentially methylated genes associated with multiple measures of AD neuropathology. Many of these genes were also differentially expressed across major brain cell types, and the results were successfully replicated in independent cohorts. Crucially, our findings suggest that DNAm may partially mediate the association between *APOE* genotype and AD pathology. These insights deepen our understanding of the role of DNAm in AD pathogenesis and may help identify potential therapeutic targets.

## Methods

### Study cohorts and participants

#### Discovery cohort

Our discovery sample included 1,250 deceased participants in five prospective cohorts at the RADC. The Religious Orders Study^17^ was initiated in 1994 and has followed older Catholic nuns, priests and brothers from across the USA since then. The Rush Memory and Aging Project^17^ started in 1997 and includes older adults from the Chicago metropolitan area. The Minority Aging Research Study^18^ was initiated in 2004, and the Rush African American Clinical Core^19^ was initiated in 2008; both the Minority Aging Research Study and the Rush African American Clinical Core recruited older African Americans from churches, senior buildings, and social clubs and organizations that serve diverse populations in the Chicago metropolitan area. The Rush Latino Core^20^ launched in 2015 and includes older adults in the Chicago area who self-identify as Latino. All studies were approved by the Institutional Review Board of Rush University Medical Center. Each participant signed an informed consent, Anatomic Gift Act, and repository consent allowing their data and biospecimens to be repurposed. A total of 1,250 DLPFC brain samples were included in the current analysis.

#### Replication cohorts

We used three independent brain DNAm datasets to replicate our findings.

1. Dataset 1 (NBB) comprised 139 DLPFC samples from the NBB, as previously described^21^. This dataset was used to replicate discovery-stage findings.
2. Dataset 2 (BSHR) comprised 127 participants from the BSHR Institute^44^. Brain tissue was collected from the inferior frontal gyrus (*n* = 117) and superior temporal gyrus (*n* = 127).
3. Dataset 3 (ROSMAP subset) comprised 100 participants independent of our discovery sample (1,250 DLPFC samples from RADC cohorts)^26^. This dataset included DLPFC (*n* = 50) and striatum (ST, *n* = 50) samples.

Datasets 2 and 3 were used to replicate associations between DNAm scores and AD pathology. All participants in replication cohorts provided written informed consent, and study procedures were approved by local institutional review boards.

### Neuropathologic assessments of AD

The RADC cohorts including ROSMAP assessed multiple AD pathologic measures including neuropathologic diagnosis of AD, Aβ load and PHFtau tangles, as previously described^45,46^. According to 2012 NIA–AA guidelines^22^, individuals received a pathologic diagnosis of no AD, low-likelihood AD, intermediate-likelihood AD or high-likelihood AD based on Thal phases of amyloid deposition, Braak NFT stage and CERAD score of neuritic amyloid plaques. Participants with an intermediate or high likelihood were classified as having a pathological diagnosis of AD; all others were classified as non-AD^22^. The evaluations were conducted by examiners blinded to all clinical data. Aβ and PHFtau tangles were quantified via immunohistochemistry across eight brain regions: the CA1–subiculum of the hippocampus, angular gyrus and entorhinal, superior frontal, mid prefrontal, inferior temporal, anterior cingulate and calcarine cortices. Mean percentage of the area positive for Aβ was averaged across brain regions. PHFtau tangle density (per mm^2^) was determined using systematic sampling; PHFtau tangle score was then averaged across brain regions.

In the NBB cohort, autopsies were performed using standardized protocols, and a final neuropathologic diagnosis (for example, AD, Parkinson’s disease or control without evidence of brain disorder) was assigned to each donor. AD diagnoses were based on clinical criteria for probable AD and confirmed histopathologically at autopsy (https://www.brainbank.nl/brain-tissue/diagnostics/#item-1). Additional neuropathologic measures, including Aβ and Braak staging, were assessed according to NIA–AA guidelines^22,47^. For replication, we included 62 AD cases and 77 controls.

In the BSHR cohort, neuropathologic AD diagnoses were determined using the NIA–Reagan criteria^48^. Participants with an intermediate or high likelihood were classified as AD.

### DNAm profiling

#### DNA extraction from postmortem brain tissue samples

In the RADC cohort, 100-mg sections of frozen DLPFC from each donor were digested in 1 ml of lysis buffer (100 mM Tris–HCl, pH 8.0; 5 mM EDTA, pH 8.0; 0.2% sodium dodecyl sulfate; 200 mM NaCl) with 30 µl of Proteinase K and incubated at 55 °C overnight. Following digestion, the lysate was added to 5 μl of RNase A and incubated in a 37 °C water bath for 1 h. One milliliter of phenol/chloroform/isoamyl alcohol (25:24:1 saturated with 10 mM Tris, pH 8.0, 1 mM EDTA) was added and mixed thoroughly before centrifugation for 10 min at 14,000 rpm. The top layer was transferred to a clean tube. Then 2 µl of glycogen (5 mg ml^−1^), 10% volume of 5 M NaCl and 2 volumes of 100% ethanol were added and mixed well. The tube was left at −20 °C overnight to precipitate DNA. The gDNA pellet was collected through spinning down at 14,000 rpm for 15 min, and then dissolved in 30 µl of nuclease-free water after washing with 70% ethanol. PicoGreen was used to quantify DNA.

#### DNAm analysis, data quality control and normalization

For the RADC samples, bisulfite conversion was completed using the EZ 96 DNA methylation Kit. About 500 ng of gDNA was used for the DNAm assay using the Infinium MethylationEPIC v2.0 BeadChip. Laboratory personnel were blinded to clinical phenotypes. Raw data were processed using the R packages minfi^49^ (v.1.50.0) and wateRmelon^50^ (v.2.10.0).

Samples were excluded if (1) the proportion of the loci detection rate was <95%, (2) the mean intensity of methylated or unmethylated signals was 3 s.d. above or below the mean, (3) bisulfite conversion efficiency was <80%, (4) there was a mismatch between reported and predicted sex, or (5) detection *P* was >0.05 in more than 1% of probes.

Probes were excluded if (1) 1% of samples had a detection *P* > 0.05, (2) beadcount was <3 in 5% of the samples, (3) the probes are on sex chromosomes, (4) probe sequences contain single nucleotide polymorphisms (SNPs) with a minor allele frequency of ≥0.05, or (5) they belong to previously reported cross-reactive probes. For probes mapping to the same genomic site, we retained the CpG with the highest quality (based on detection *P* values and bead counts). Non-CpG sites were excluded from further analysis.

The subsequent normalization of DNAm data was first performed using a quantile normalization method implemented by the ‘dasen’ function in the R package wateRmelon. We then applied the beta-mixture quantile normalization approach implemented in the R package wateRmelon to normalize beta values of type I and type II design probes. Given that methylation *M* values (logit-transformed methylation *β* values) have better statistical properties (that is, homoscedasticity) for linear regression models, we derived *M* values for the downstream analysis. We used the R package SVA^24^ (v.3.46.0) to detect and estimate surrogate variables for unknown sources of variation to remove artifacts from the high-throughput experiments. In line with previous work, we adjusted for a variable number of surrogate variables until inflation index (*λ*) fell below 1.2. We estimated the proportion of NeuN-positive cells in each sample using reference data from flow sorted frontal cortex cell populations^51^ implemented in the R package minfi.

In the NBB cohort, gDNA was extracted from brain tissue samples using the Qiagen AllPrep DNA/RNA/miRNA Universal Kit, bisulfite-converted and assayed on the Illumina Infinium MethylationEPIC v1 array (>850,000 CpGs), as previously described^23^. Clinical and DNAm data were obtained from GEO (GSE203332) and processed following the same procedures described above. A total of 139 DLPFC samples were included in the replication analysis.

After stringent quality control and normalization (Extended Data Fig. 1), the discovery dataset included 875,741 autosomal probes from 1,250 individuals. The NBB replication dataset contained 807,422 autosomal probes from 139 unique individuals. A total of 696,281 probes overlapping between cohorts (1,250 in RADC; 139 in NBB) were retained for replication analysis.

To further replicate the associations between DNAm scores and AD neuropathology, we analyzed two independent cohorts: the BSHR cohort (117 in inferior frontal gyrus, 127 in superior temporal gyrus) and the ROSMAP subset (50 in DLPFC, 50 in striatum). In both cohorts, gDNA was extracted from brain tissue, bisulfite-converted and assayed using the Illumina Infinium MethylationEPIC v1 array (>850,000 CpGs), as previously described^25,26^. Processed DNAm data were obtained from GEO (GSE156984) for BSHR and from Synapse (syn23633755) for the ROSMAP subset. In both datasets, *β* values were converted to *M* values for association analysis. Demographic characteristics of participants in replication cohorts (NBB, BSHR, ROSMAP subset) are summarized in Supplementary Table 1.

#### *APOE* genotyping

*APOE* genotypes in the RADC cohort were determined by sequencing codons 112 and 158 of the *APOE* gene in DNA extracted from either peripheral blood or frozen postmortem brain tissue^52^. *APOE* ε4 carriers (*n* = 277) were defined as individuals with either one or two copies of the ε4 allele (ε4/ε4, ε3/ε4). To avoid a potential confounding effect by the ε2 allele, we excluded *APOE* ε2/ε4 carriers (*n* = 28) from the mediation analysis. *APOE* genotypes were not available in the replication cohorts (NBB, BSHR).

#### Brain snRNA-seq data in RADC

Brain (DLPFC) snRNA-seq data in RADC were downloaded from Synapse (https://www.synapse.org/Synapse:syn31512863). Detailed methods for snRNA-seq data generation and preprocessing have been described previously^53,54^. Briefly, nuclei were isolated from 479 DLPFC human brain tissues. Each library-construction batch included nuclei suspensions from eight participants, except B63, which included seven. In each batch, nuclei were pooled and processed using the 10X Genomics 3 Gene Expression kit (v3 chemistry). The libraries were sequenced, and read mapping and unique molecular identifier (UMI) counting were performed using CellRanger software v.6.0.0 (10X Genomics) with GENCODE v.32 and GRCh38.p13. As a quality control, genotype concordance of RNA and whole-genome sequencing, sex check, duplicated participants, whole-genome sequencing quality control, and sequencing depth were assessed; 437 participants passed the quality control. Using the cell-type annotations of previous work^55^, a regularized logistic regression classifier was constructed to infer cell types based on single-nucleus gene expression. This classifier was applied to each cell of single-nucleus libraries. Low-quality nuclei were then determined using cell-type-specific thresholds of total number of UMIs and number of unique features, and low-quality nuclei were removed. Doublets were detected by running DoubletFinder^56^. A pseudobulk UMI count matrix for each cell type was generated by extracting UMI counts of the cell type and by aggregating the counts per gene per individual. Low-expression genes were filtered out using filterByExpr function in the Bioconductor package edgeR^57^ with its default parameters. Pseudobulk counts were normalized by using the trimmed mean of *M* values method in the Bioconductor package edgeR, and log_2_ of counts per million mapped reads were computed using the voom function in the Bioconductor package limma^58^. All 437 participants with snRNA-seq data also had DNAm data. Our analyses focused on seven major cell type groups: excitatory neurons, inhibitory neurons, astrocytes, microglia (which included monocytes and macrophages), oligodendrocytes, oligodendrocyte precursor cells and vascular niche (which included endothelial cells, pericytes, arterial smooth muscle cells and fibroblasts).

### Statistics and reproducibility

All analyses were performed using the R program in RStudio (v.4.2.3). Benjamini–Hochberg adjusted FDR <0.05 was considered significant.

#### Epigenome-wide association analysis

We performed EWAS to examine the association of individual CpG sites with each of the three AD neuropathological measures—AD diagnosis, Aβ load and PHFtau tangles—in the RADC cohort (*n* = 1,250). The DNAm level (*M* value) at each probe was regressed against each neuropathologic measure using linear regression, adjusting for age at death, sex, race (white or nonwhite), PMI, proportion of NeuN-positive cells, experiment plates and unwanted variation characterized by the first three surrogate variables derived using the R package SVA^24^. The distribution of *M* values (logit-transformed methylation *β* values) was assumed to be approximately normal, but this was not formally tested. Top hits were then replicated in the NBB cohort (*n* = 139), with replication defined as CpG probes showing FDR < 0.05 and a consistent direction of effect in both cohorts. EWAS results from both cohorts were processed using Bacon correction via the R package bacon (v.1.26.0)^59^, a Bayesian method designed to control for inflation in EWAS. Multiple testing in the RADC discovery sample accounted for 875,741 CpGs. By contrast, replication analysis in the NBB accounted for only a subset of CpGs that were significant in the discovery phase and also available in the NBB dataset.

To evaluate whether the identified DMPs distinguish individuals with and without neuropathological AD, we constructed a DNAm score as the weighted sum of DMPs replicated in NBB, using regression coefficients from EWAS of the discovery phase as weights. This DNAm score was further assessed in two independent brain datasets, BSHR (inferior frontal gyrus, *n* = 117; superior temporal gyrus, *n* = 127) and ROSMAP (DLPFC, *n* = 50; striatum, *n* = 50).

#### DMR analysis

DMRs associated with each neuropathological measure were identified using the software comb-p^27^. *P* values from the RADC discovery sample served as input. Putative DMRs were defined as regions containing two or more CpG sites with EWAS *P* < 10^−4^ with a 500 bp window. Following multiple testing correction implemented in comb-p, a DMR with a Sidak-adjusted family-wise error rate <0.05 was considered statistically significant^27^.

#### Mediation analysis

To examine whether DNAm mediates the effect of *APOE* ε4 allele (exposure) on neuropathologic AD diagnosis (outcome), we first tested the association between the identified DMPs (confirmed in both the RADC discovery and NBB replication cohorts) and the *APOE* ε4 allele, and then constructed a DNAm score using these *APOE*-associated DMPs, weighted by regression coefficients obtained from the discovery sample. Next, we regressed out covariates (age at death, sex, race (white or nonwhite), PMI, proportion of NeuN-positive cells, experiment plate and the first three surrogate variables) from the DNAm score and used the residual (mediator) in the mediation analysis. The analyses were performed using the R package mediation (v.4.5.0) to estimate the direct, indirect (mediated) and total effects^28^. The direct effect represented the effect of exposure on outcome that was independent of DNAm (mediator). The indirect effect represented the effect of exposure on the outcome that could be explained by DNAm (mediator). The total effect of exposure on outcome includes both the direct effect and the indirect effect via the mediator. The proportion mediated by the mediator was defined as (indirect effect/total effect) × 100%. *P* values and CIs were derived by the quasi-Bayesian approximation with 1 × 10^6^ Monte Carlo simulations. Furthermore, we applied the same method to assess the individual mediating effect of each *APOE*-associated DMP on the association between *APOE* genotype and neuropathologic diagnosis of AD. This analysis included 1,183 RADC participants with available *APOE* genotype data; it was performed in the RADC discovery sample only because *APOE* genotype data were not available in other replication cohorts.

#### Gene annotation

Gene annotations for the identified DMPs were performed using the Genomic Regions Enrichment of Annotations Tool (GREAT)^60^ (v.4.0.4), which annotates a DMP to genes with the TSS within 5 kb upstream or 1 kb downstream, with extension up to 1 Mb.

#### Cell-type-specific gene expression analysis

To examine the potential functional impact of the identified DMPs in the EWAS, we investigated whether DMP-annotated genes identified in the bulk DLPFC exhibited differential expression in major brain cell types using snRNA-seq data from the same cortex of the same individuals in the RADC (*n* = 437)^53,54^. The cell-type-specific expression of each candidate gene was regressed against each AD neuropathologic measure using linear regression, adjusting for age at death, sex, PMI and batch effects.

#### Methylation quantitative trait loci mapping in the RADC cohort

To perform colocalization analysis, we first performed methylation quantitative trait loci (mQTL) mapping using 773 RADC participants who had both DNAm data (PFC brain region, measured by Infinium MethylationEPIC v2 array) and whole-genome sequencing data^61^. Linear regression models were constructed to test the association between each CpG of interest and its *cis*-SNPs (±50 kb). The analyses were adjusted for age at death, gender, PMI, the first ten genetic PCs and the first five methylation PCs. An SNP was considered as a significant mQTL for the specific CpG site if the Benjamini–Hochberg-adjusted *P* value (FDR) < 0.05.

#### Colocalization analysis

To investigate whether DMPs and the annotated genes share common causal variants, we performed colocalization analysis using the R package coloc^29^. The program estimates the posterior probability of a single variant affecting both DNAm and gene expression. We first performed mQTL mapping for AD neuropathology-associated DMPs using the whole-genome sequencing data generated in the RADC cohort (*n* = 773). We then conducted colocalization analysis using *cis*-mQTL results in the RADC cohort and cell-type-specific expression quantitative trait loci (eQTL) summary statistics (https://zenodo.org/records/7276971) in eight human brain cell types in PFC, temporal cortex and white matter^62^. We used the default prior probabilities that a variant is equally associated with each molecular phenotype (*P*_1_ = 1 × 10^−4^; *P*_2_ = 1 × 10^−4^) and both molecular phenotypes jointly (*P*_12_ = 1 × 10^−5^). A posterior probability of the DNAm and expression sharing a common variant >0.8 (*PP*_4_ > 0.8) indicates the significant evidence of colocalization. This analysis focused on AD pathology-associated DMPs identified in the RADC discovery sample (FDR < 0.05).

#### Enrichment analysis

We used GARFIELD v.2.0^30^ to assess enrichment of AD neuropathology-associated DNAm sites in functional and regulatory genomic elements by integrating 1,005 annotations from ENCODE, GENCODE and the Roadmap Epigenomics Project. Enrichment significance was assessed by permutation while accounting for minor allele frequency, the number of linkage disequilibrium proxies (*r*^2^ >0.8), and distance to the nearest TSS. GWAS summary statistics from a large-scale meta-analysis were used^31^.

For methylation sites of interest, we curated a list of genetic variants comprising *cis*-mQTLs associated with these CpGs and *cis*-eQTLs associated with the CpG-annotated genes. The mQTLs were obtained from either mQTL mapping in the RADC brain samples or previous large-scale blood-based mQTL studies^63–65^, while eQTLs were derived from prior brain transcriptomic studies^66,67^. We performed enrichment analysis by GARFIELD using this list of genetic variants under different GWAS *P* value thresholds (1 × 10^−4^, 1 × 10^−5^, 1 × 10^−6^, 1 × 10^−7^, 1 × 10^−8^). *P* values of the enrichment analysis were adjusted for multiple testing based on the effective number of annotations. This analysis focused on 3,338 DNAm sites with *P* < 5 × 10^−4^ in the RADC discovery sample.

### Reporting summary

Further information on research design is available in the Nature Portfolio Reporting Summary linked to this article.

## Supplementary information

Reporting Summary

Peer Review File

Supplementary Tables 1–10. Excel file containing Supplementary Tables 1–10 (each under a separate tab). The legend of each table is included in each tab.

## Source data

Source Data Fig. 1 Statistical source data.

Source Data Fig. 2 Statistical source data.

Source Data Fig. 3 Statistical source data.

Source Data Extended Data Fig. 4 Statistical source data.

Source Data Extended Data Fig. 6 Statistical source data.

Source Data Extended Data Fig. 7 Statistical source data.

Source Data Extended Data Fig. 8 Statistical source data.

Source Data Extended Data Fig. 9 Statistical source data.

## Data Availability

Brain DNA methylation (DNAm) data (DLPFC region, measured using the Infinium MethylationEPIC v2 array) and corresponding phenotype data from the Rush Alzheimer’s Disease Center (RADC) cohorts are available to qualified researchers upon request via the RADC Resource Sharing Hub (https://www.radc.rush.edu/). Access to these datasets requires submitting a formal data request application and signing a Data Use Agreement (DUA) through the RADC Resource Sharing Hub administrator. Brain snRNA-seq data from the RADC is available at Synapse (syn31512863). Brain DNAm data (DLPFC region, measured by Infinium MethylationEPIC array) from the Netherlands Brain Bank (NBB) study is available on the Gene Expression Omnibus (GEO) under accession number GSE203332. Brain DNAm data (samples from DLPFC and striatum regions, measured by Infinium MethylationEPIC v1 array) from the ROSMAP is available at Synapse (syn23633755). Brain DNAm data (samples from inferior frontal gyrus and superior temporal gyrus regions, measured by Infinium MethylationEPIC array) from the Banner Sun Health Research (BSHR) is available on the GEO under accession number GSE156984. Source data are provided with this paper.

## Code availability

The code used to perform the analyses described in this study is available via GitHub at https://github.com/Zhao-research/Brain-DNAm and via Zenodo at https://doi.org/10.5281/zenodo.20558402 (ref. ^68^).
