# Supplementary material for: Brain DNA methylation, APOE genotype and Alzheimer’s disease neuropathology in a large postmortem cohort
Source: Nat Aging. 2026 Oct 12;6(11):2493–503. doi: 10.1038/s43587-026-01243-7 (PMC13754617; doi:10.1038/s43587-026-01243-7)
Supplement: Supplementary file 1 [file 43587_2026_1243_MOESM1_ESM.pdf]

nature portfolio

Corresponding author(s): Jinying Zhao, MD, PhD - Jinying.Zhao@epi.usf.edu

Last updated by author(s): 08/12/2026

Reporting Summary

Nature Portfolio wishes to improve the reproducibility of the work that we publish. This form provides structure for consistency and transparency in reporting. For further information on Nature Portfolio policies, see our Editorial Policies and the Editorial Policy Checklist.

Statistics

For all statistical analyses, confirm that the following items are present in the figure legend, table legend, main text, or Methods section.

n/a	Confirmed	
☐	☒	The exact sample size (n) for each experimental group/condition, given as a discrete number and unit of measurement
☐	☒	A statement on whether measurements were taken from distinct samples or whether the same sample was measured repeatedly
☐	☒	The statistical test(s) used AND whether they are one- or two-sided<br>*Only common tests should be described solely by name; describe more complex techniques in the Methods section.*
☐	☒	A description of all covariates tested
☐	☒	A description of any assumptions or corrections, such as tests of normality and adjustment for multiple comparisons
☐	☒	A full description of the statistical parameters including central tendency (e.g. means) or other basic estimates (e.g. regression coefficient) AND variation (e.g. standard deviation) or associated estimates of uncertainty (e.g. confidence intervals)
☐	☒	For null hypothesis testing, the test statistic (e.g. F, t, r) with confidence intervals, effect sizes, degrees of freedom and P value noted<br>*Give P values as exact values whenever suitable.*
☐	☒	For Bayesian analysis, information on the choice of priors and Markov chain Monte Carlo settings
☐	☒	For hierarchical and complex designs, identification of the appropriate level for tests and full reporting of outcomes
☐	☒	Estimates of effect sizes (e.g. Cohen's d, Pearson's r), indicating how they were calculated

Our web collection on statistics for biologists contains articles on many of the points above.

Software and code

Policy information about availability of computer code

Data collection	About 500 ng gDNA from each RADC participant was bisulfite treated and assessed using the Infinium HumanMethylationEPIC v2.0 BeadChip (Illumina) following the manufacture's protocol.
Data analysis	DNA methylation pre-processing: R-packages minfi v1.50.0, wateRmelon v2.10.0<br>Downstream analyses: R-packages SVA v3.46.0, bacon v1.26.0, mediation v4.5.0<br>Differentially methylated region (DMR) analysis: comb-p software v0.50.6<br>Colocalization analysis: R-package coloc v5.2.3<br>Functional enrichment analysis: GARFIELD v2<br>The code used to perform the analyses described in this study is available on GitHub at https://github.com/Zhao-research/Brain-DNAm and Zenodo at https://doi.org/10.5281/zenodo.20558402.

For manuscripts utilizing custom algorithms or software that are central to the research but not yet described in published literature, software must be made available to editors and reviewers. We strongly encourage code deposition in a community repository (e.g. GitHub). See the Nature Portfolio guidelines for submitting code & software for further information.

Data

Policy information about availability of data

All manuscripts must include a data availability statement. This statement should provide the following information, where applicable:

- Accession codes, unique identifiers, or web links for publicly available datasets
- A description of any restrictions on data availability
- For clinical datasets or third party data, please ensure that the statement adheres to our policy

Brain DNA methylation (DNAm) data (DLPFC region, measured by Infinium MethylationEPIC v2 array) and phenotype data from the Rush Alzheimer's Disease Center (RADC) cohorts are available at https://www.radc.rush.edu/. Brain snRNA-seq data from the RADC is available at Synapse (syn31512863). Brain DNAm data (DLPFC region, measured by Infinium MethylationEPIC array) from the Netherlands Brain Bank (NBB) study is available on the Gene Expression Omnibus (GEO) under accession number GSE203332. Brain DNAm data (samples from DLPFC and striatum regions, measured by Infinium MethylationEPIC array) from the ROSMAP is available at Synapse (syn23633755). Brain DNAm data (samples from inferior frontal gyrus and superior temporal gyrus regions, measured by Infinium MethylationEPIC array) from the Banner Sun Health Research (BSHR) is available on the GEO under accession number GSE156984.

Research involving human participants, their data, or biological material

Policy information about studies with human participants or human data. See also policy information about sex, gender (identity/presentation), and sexual orientation and race, ethnicity and racism.

Reporting on sex and gender	Sex was self-reported. Sex was included as a covariate in all analyses.
Reporting on race, ethnicity, or other socially relevant groupings	Among 1,250 RADC participants included in the downstream analysis, 1,166 are non-Latinx Whites, 66 are African-American, 5 is Native American, and 13 belong to other racial groups.
Population characteristics	Covariates included age at death, sex, race (Whites or non-Whites), postmortem interval (PMI), proportion of NeuN positive cells, and experiment plate. Table 1 summarizes characteristics of the study participants.
Recruitment	The RADC discovery sample included deceased participants in studies by the RADC from 5 prospective cohorts, including ROS, MAP, MARS, AA Core, and LATC. All participants without known dementia were enrolled, received annual clinical evaluations, and agreed to donate their brains at the time of death.
Ethics oversight	All aspects of the RADC studies were approved by an Institutional Review Board of Rush University Medical Center. Each participant signed an informed consent, Anatomic Gift Act, and repository consent allowing their data and biospecimens to be repurposed.

Note that full information on the approval of the study protocol must also be provided in the manuscript.

Field-specific reporting

Please select the one below that is the best fit for your research. If you are not sure, read the appropriate sections before making your selection.

☒ Life sciences ☐ Behavioural & social sciences ☐ Ecological, evolutionary & environmental sciences

For a reference copy of the document with all sections, see nature.com/documents/nr-reporting-summary-flat.pdf

Life sciences study design

All studies must disclose on these points even when the disclosure is negative.

Sample size	We did not perform a sample size calculation a priori. The analysis included the largest sample size available, including participants who had both brain DNA methylation and neuropathologic data (total N= 1,250). For other omics data, sample size was chosen based on data availability.
Data exclusions	Samples were excluded if 1) the proportion of loci detection rate <95%, 2) the mean intensity of methylated or unmethylated signals were three standard deviations above or below the mean, 3) the bisulfite conversion efficiency <80%, 4) there was a mismatch between reported and predicted sex, or 5) the detection P>0.05 in more than 1% of probes. DNA methylation probes were excluded if 1) 1% of samples with a detection P>0.05, 2) the beadcount <3 in 5% of samples, 3) the probes are on sex-chromosomes, 4) probe sequences contain SNPs with minor allele frequency (MAF) ≥0.05, or 5) they belong to previously reported cross-reactive probes.
Replication	We used the 1,250 RADC samples for discovery and replicated our findings in 3 independent datasets: 1) 139 brain samples from the Netherlands Brain Bank (NBB), 2) 244 brain samples from the Banner Sun Health Research (BSHR) Institute, 3) 100 brain samples for the Religious Orders Study and Memory and Aging Project (ROSMAP) cohort.
Randomization	This was an observational prospective cohort study with no experimental group assignment or intervention; therefore, randomization was not applicable.

Blinding	The individuals preparing samples were blinded to all phenotypic information. This is not a clinical trial so blinding is not applicable to the study.

Reporting for specific materials, systems and methods

We require information from authors about some types of materials, experimental systems and methods used in many studies. Here, indicate whether each material, system or method listed is relevant to your study. If you are not sure if a list item applies to your research, read the appropriate section before selecting a response.

Materials & experimental systems

n/a	Involved in the study
☒	☐ Antibodies
☒	☐ Eukaryotic cell lines
☒	☐ Palaeontology and archaeology
☒	☐ Animals and other organisms
☒	☐ Clinical data
☒	☐ Dual use research of concern
☒	☐ Plants

Methods

n/a	Involved in the study
☒	☐ ChIP-seq
☒	☐ Flow cytometry
☒	☐ MRI-based neuroimaging

Plants

Seed stocks	*Report on the source of all seed stocks or other plant material used. If applicable, state the seed stock centre and catalogue number. If plant specimens were collected from the field, describe the collection location, date and sampling procedures.*
Novel plant genotypes	*Describe the methods by which all novel plant genotypes were produced. This includes those generated by transgenic approaches, gene editing, chemical/radiation-based mutagenesis and hybridization. For transgenic lines, describe the transformation method, the number of independent lines analyzed and the generation upon which experiments were performed. For gene-edited lines, describe the editor used, the endogenous sequence targeted for editing, the targeting guide RNA sequence (if applicable) and how the editor was applied.*
Authentication	*Describe any authentication procedures for each seed stock used or novel genotype generated. Describe any experiments used to assess the effect of a mutation and, where applicable, how potential secondary effects (e.g. second site T-DNA insertions, mosiacism, off-target gene editing) were examined.*
