# Supplementary material for: Brain DNA methylation, APOE genotype and Alzheimer’s disease neuropathology in a large postmortem cohort
Source: Nat Aging. 2026 Oct 12;6(11):2493–503. doi: 10.1038/s43587-026-01243-7 (PMC13754617; doi:10.1038/s43587-026-01243-7)
Supplement: Supplementary file 2 [file 43587_2026_1243_MOESM2_ESM.pdf]

nature portfolio

Peer Review File

Brain DNA methylation, APOE genotype, and AD neuropathology: findings from a large cohort of postmortem human brain tissue samples

Corresponding Author: Dr Jinying Zhao

Version 0:

Reviewer comments:

Reviewer #1

(Remarks to the Author)
This paper represents an important step in studying the role of methylation in AD. It expands on the number of probes examined relative to earlier studies in a large post-mortem brain tissue sample. It also has the advantage of a replication sample that supports many of the associations observed. They examined methylation in relation to AD pathology, Aβ, and PHFtau. Several of these loci co-localize with known genetic risk variants. I only have a few concerns about how the data are presented and how the results are interpreted.

Concerns:
The ROC analysis may be overly optimistic if it was fitted on sites that were already significant in both the training and the replication dataset. Additionally, the clinical utility of a biomarker that relies on the sampling of brain tissue is unclear, and I have a hard time seeing how this can be used to identify those at high and low AD risk as stated in the discussion.

In Figure 3a, it is clear that the scores would have to differ between cases and controls, as it is computed strictly from DMP that were identified in these datasets. It isn't clear whether or not these scores would generalize to other datasets, or whether the observed difference would be so pronounced.

In the mediation analysis, the score is computed from AD-associated probes, and this score is tested as a mediator of the effect of the APOE genotype. This brings up the question as to whether the included DMP were APOE associated, AD associated, or both. Some examination of the APOE-associated vs. non-APOE-associated positions is warranted. It would also be worth commenting in the main text as whether or not any associated probes are in the APOE region itself (cis). From the supplement, the probes included in the score are for the most part not on chromosome 19. Do you expect so much trans regulation of DNA methylation based on prior studies of APOE and methylation?

The EPIC V2 array has multiple probes assessing the same position. Was this accounted for in the EWAS and DMR analysis?

In several places, the paper states that DNAm mediates the APOE effect. It would be better to state that it mediates a portion of the APOE effect throughout, to avoid giving the impression that methylation in the PFC explains the whole APOE effect on AD risk. It is worth noting in the discussion that when multiple brain regions are taken into account that the proportion of mediated variation could be higher.

Reviewer #2

(Remarks to the Author)
This study presents a large-scale epigenome-wide association analysis of Alzheimer's disease (AD) neuropathology, integrating Illumina MethylationEPIC v2 arrays in a discovery cohort (n=1,250) with a smaller replication cohort (n=139), and further linking findings to single-nucleus RNA sequencing (snRNA-seq) data in a subset of the discovery cohort. The manuscript offers valuable insights into potential differentially methylated sites that might mediate known genetic risk (APOE). Overall, the study is timely and valuable to the neurodegenerative disease research community. However, several

concerns and points need to be addressed.

Major comments:

1. In addition to DMP analysis, the authors should also perform DMR (differentially methylated regions) analysis, which reduces influence of single-site noise, and often align more with biological meaningful features.

2. Please clarify the intended clinical utility of the methylation risk score and address the possibility of over-fitting. The 38 neuropathology-associated DMPs were discovered, replicated, and then reused to build risk models in the same two cohorts, an approach that can inflate performance estimates. In addition, an AUROC of ~0.72 for a model evaluated on its own training data appears low, and it is counter-intuitive that the AUROC is higher in the test set than in the training set.

3. Because the prediction analyses were performed using brain samples, the translational value of the findings would increase if cross-tissue concordance and external performance in blood samples are assessed. Therefore, please quantify brain-to-blood correlations for the 38 replicated DMPs, construct a peripheral-blood methylation score using the CpGs that show robust concordance, and then evaluate that score in an independent blood DNA-methylation dataset (e.g., ADNI) to demonstrate its potential utility for risk stratification in living individuals.

4. Table 2 is currently missing. Please provide it and for each of the 38 replicated DMPs, add two annotations:
(1) Novelty status - Indicate whether the CpG has been reported in any previous brain EWAS of AD-related neuropathology.
(2) Array coverage - State explicitly whether the probe is present on the older Illumina 450 K array.
These columns will let readers see at a glance whether a CpG is "novel" because it was not significant in earlier 450 K analyses or because it was never assayed on the 450 K platform in the first place, and will highlight the findings most worthy of further follow-up.

5. In the mediation analysis, the authors computed "The DNAm score considers the association direction of 38 DMPs with AD neuropathology, which is defined as the sum of M-values of 10 DMP_pos minus Mvalues of 28 DMP_neg". Please explain why the methylation score was defined as the un-weighted sum of CpG M-values. Most methylation risk scores weight each CpG by its EWAS effect size (β-coefficient), see Huls et al., 2020 (PMID 31318318), to reflect the relative contribution of each probe. Please recompute the score using such weights or justify why an un-weighted approach is appropriate, and report whether the mediation results still holds.

6. In addition, please consider performing single-CpG mediation tests to identify which of the 38 DMPs drive the aggregate effect.

7. In the Methods section, the authors described two different meta-analytic frameworks: a random-effects model for the RADC + NBB analysis ("Meta-analysis") and a fixed-effects model for the three external DLPFC EWAS ("Comparison with previous EWAS results"). Please explain this discrepancy and consider applying the same model to both analyses.

8. Also, please clarify the added value of the meta-analysis of the ROSMAP, London, and Mount Sinai DLPFC datasets when these cohorts have already been meta-analysed in prior publications. It's not clear why existing meta-analytic results were insufficient.

8. Please add a discussion comparing your findings with those of Shireby et al. 2022 (PMID 36153390), which mapped DMPs in purified brain cell types.

9. Please clarify why the enrichment was performed on GWAS variants that merely harbor the neuropathology-associated CpGs and why a nominal threshold of $P < 1 \times 10^{-4}$ was chosen. A more biologically coherent strategy would be to base the test on (i) cis- or trans-mQTLs that regulate the significant CpGs, (ii) eQTLs or expression traits linked to those CpGs, or (iii) the CpGs themselves.

10. Please comment on how the findings overlap across the three neuropathologic phenotypes. Discuss what this pattern of overlap, or lack thereof, suggests about common versus trait-specific molecular mechanisms.

Minor comments:

1. Please supply the Supplementary Tables as Excel (.xlsx) files so that they are easier to read and reuse by other researchers.

2. On line 70, add one sentence about how neuropathologic diagnosis of AD was defined.

3. In Methods, it was mentioned "DNAm level at each probe was regressed against each AD neuropathologic measure using linear regression". Please clarify was methylation beta values or M-values were used as outcome?

4. In Table 1, tabulate race and ethnicity distribution of the subjects

5. In legends of Figure 3, clarify which dataset was analyzed.

6. In Figure 3, provide exact values for P-value

7. On line 179, start a new paragraph

Reviewer #3

(Remarks to the Author)
Miao and colleagues present an interesting manuscript describing a DNA methylation, APOE genotype and AD neuropathology study on a large cohort of human postmortem brain tissue. The major novelties of this study are: 1) the use of the Illumina EPIC arrays V2, which have much higher coverage of methylation sites throughout the genome compared to the 450K arrays used in most of the previous large AD studies, and 2) the analysis considering the APOE genotype. Overall, this paper reports interesting findings, which can contribute to the advancement of knowledge regarding epigenetics of AD. However, I have identified several issues with room for improvement, which are listed below.
1) The introduction is very brief (seems shorter than the abstract). It would be good to have a bit more context of previous findings from the largest AD studies, some of which looked not only at AD status, but also tau and amyloid pathology (e.g. Shireby et al 2022) and this should be made clear. Additionally, the authors should at least highlight DNA methylation changes in AD brain tissue studies that have been consistently reported across studies.
2) In the results, it is not clear how the neuropathological diagnosis of AD was established and this is important for the interpretation of the findings and cross-study comparisons. This should therefore be made clear.
3) Table 2 is missing and it is therefore not possible to assess the results that should be shown there.
4) In the general description of the EWAS findings (page 4), it is interesting to note the newly discovered associations. However, it would be good to see a bit more on description/discussion of the overlap with findings from previous AD EWAS and large meta-analyses. The 11 CpGs reported in eTable 4 and 7 CpGs in eFigure 4 seem quite a limited replication of previous findings, which is concerning. It is not even clear whether the direction of effect is the same as previously reported or not, which could mean even less overlap, and this should therefore be clarified. Wouldn't the authors expect more extensive overlap with previous studies (at least the larger ones)? It doesn't seem to be fully explained by the use of different arrays in the different studies as the new EPIC arrays are overall more comprehensive than the previous 450K not less. Please elaborate on the reasons for this very limited overlap.
5) For the AD predictions using DNAm features (page 5), would it improve predictions if using all the CpG from the meta-analysis rather than the 38 DMPs found in both discovery and replication cohorts? As the authors also looked at APOE status in this study, would the APOE status add anything to the AD predictions?
6) Does the DNAm score composed of the 38 DMPs differ between APOE4 carriers and non-carriers?
7) Regarding the cell-type specific expression, eFigure 5 is incomplete (right end seems to be cut), and it should be explained what is represented in each of the three panels.
8) In the genetic co-regulation and enrichment analysis (page 6), why was the GWAS threshold set to 1x10-4?
9) In the discussion (page 7, lines 144 to 149), there are several broad statements referring to "many genes", "several genes", "novel genes… many of which", it would be useful to have specific figures/numbers for each of these statements.
10) Also in the discussion (page 8, lines 175-6), given that this study investigated brain tissue, which is not easily accessible in living patients, I would suggest to tone down the statement saying these findings "…may have potential value in risk stratification…", and recommend caution in interpretation without further investigation in accessible tissues.
11) In the supplementary content, in page 7, it is not clear how many tests were considered for the FDR correction, was it genome-wide for both discovery and replication cohort? (It is noted that for NBB, in eTable 2, unadjusted and FRD adjusted p-values are quite similar). In page 9, why were cellular proportions (major contributors to variance in DNAm) not included in the regression models for the additional datasets? In page 11, it is not clear how mQTLs were generated. Also, it should be acknowledged that the datasets used for enrichment analyses (page 12), such as those used by GARFIELD, which are not brain related (eTable 9), and the Bellenguez et al. GWAS, which is not a pure AD GWAS (as it includes proxy cases, for example), have limitations and should be interpreted with caution.

Version 1:

Reviewer comments:

Reviewer #2

(Remarks to the Author)
I thank the authors for thoroughly addressing my concerns and comments.

Reviewer #4

(Remarks to the Author)
This is a worthwhile manuscript and it recieved a throrugh review in the first round. I agree with the first reviewer's points and I think that in the main the authors have adequately dealt with them. This includes substantive points from reviewers 1 and 3.

I could of course come up with a few additional points but I am resisting because I don't think this is a good point to make additional demands, but I will allow myself to two simple points:
1) in the methods the preprocessing is described as being QN followed by BMIQ using wateRmelon. WateRmelon has a number of QN based methods, the authors should say which one they did use.
2) from github I can see the answer to 1 is Dasen. The code availability is exemplary. The authors should include a download to the repo in supplementary materials to give easy, guaranteed access to the code.

Reviewer #5

(Remarks to the Author)
The authors have provided a systematic response to the comments from the three reviewers, and have implemented a number of changes to the manuscript. I have only a few minor comments, in response to the amendments made.

Reviewer 2 - comment 11 - amendments to manuscript on page 12. The authors have added to the discussion in the revised manuscript, reflecting on the CpGs uniquely associated with different phenotypes. The authors comment that one CpG (cg00744233) was exclusively associated with overall pathological AD diagnosis [so not with amyloid or tau], and also about others that are associated with more than one trait. However, in reality, what they are referring to here is possibly reflecting this CpG meeting the Bonferroni significance threshold in only this analysis [but it may be showing pathology-associated changes just missing significance in the other regions]. The authors should explore for each of the 38 DMPs whether they were showing a similar pattern in the other phenotypes (ie is the direction of effect the same as expected, and whether these were at least nominally significant) to be able to claim CpGs are exclusively unique. Effect size and p value for the other comparisons should be added to the relevant supplementary tables for the analysis of each phenotype, and this reflected on in the discussion, if seen.

Reviewer 3 - comment 1 (changes page to manuscript in pages 3-4). The authors have added a nice background about other studies in this area. There is one slightly misleading statement. In line 62 the authors say "CpG sites within several gene cluster, such as ANK1, RHBDF2 and HOX". However, the previous EWAS in field have not seen changes in ANK1 or RHBDF2 gene clusters, just in these genes. In addition, brain EWAS have highlighted the HOXA cluster (whilst the HOXB has been reported in blood. The authors should rephrase this to say "CpG sites within several genes, such as ANK1, RHBDF2 and the HOXA gene cluster"

#Reviewer 3 - comment 11 - changes to manuscript on page 9 line 202-205. The authors add details about the results of this analysis, however these are vague and simply state they saw significant enrichments. Can they state here in the results what these were]

Amendments to page 13 - lines 280-284. The addition of this section has made the next sentence confusing "Future research should incorporate single-cell technologies and examine additional brain regions, as in a recent study, despite its smaller sample size" Can the authors clarify what they are referring to by "despite its smaller sample size".

I have one point relating to the meta-analyse of the two cohorts (RADC and NBB). Generally meta-analyses in just two cohorts are avoided, particularly if one study is a lot larger (which is the case here) as it can essentially influence the pooled effect size, which will be driven by the larger cohort. what was the rationale for meta-analysis, rather than simply replication?

I have another query about how adjusted for race in the EWAS - it looks like the linear models simply used a binary variable (white vs non-white). was there a reason why the authors did not use genetic PCs to account for ethnicity?

Version 2:

Reviewer comments:

Reviewer #5

(Remarks to the Author)
Thank you to the authors for carefully considering the points of the reviewers. In my opinion, these have all been addressed in the updated manuscript.

Open Access This Peer Review File is licensed under a Creative Commons Attribution 4.0 International License, which permits use, sharing, adaptation, distribution and reproduction in any medium or format, as long as you give appropriate credit to the original author(s) and the source, provide a link to the Creative Commons license, and indicate if changes were made.
In cases where reviewers are anonymous, credit should be given to 'Anonymous Referee' and the source.
The images or other third party material in this Peer Review File are included in the article's Creative Commons license, unless indicated otherwise in a credit line to the material. If material is not included in the article's Creative Commons license and your intended use is not permitted by statutory regulation or exceeds the permitted use, you will need to obtain permission directly from the copyright holder.
To view a copy of this license, visit https://creativecommons.org/licenses/by/4.0/

Response to reviewers' comments

We thank the editor and reviewers for their thoughtful evaluation of our manuscript and for the opportunity to submit a revision. As requested, we have provided a revised manuscript (with changes highlighted in blue) along with a detailed, point-by-point response below. Please note that all line numbers, as well as the order of tables and figures, refer to the revised version. Authors' responses are indicated in blue.

REVIEWER COMMENTS

Reviewer #1 (Remarks to the Author):

This paper represents an important step in studying the role of methylation in AD. It expands on the number of probes examined relative to earlier studies in a large post-mortem brain tissue sample. It also has the advantage of a replication sample that supports many of the associations observed. They examined methylation in relation to AD pathology, Aβ, and PHFtau. Several of these loci co-localize with known genetic risk variants. I only have a few concerns about how the data are presented and how the results are interpreted.

Response: We greatly appreciate the reviewer's positive comments on our manuscript.

Concerns:

1. The ROC analysis may be overly optimistic if it was fitted on sites that were already significant in both the training and the replication dataset. Additionally, the clinical utility of a biomarker that relies on the sampling of brain tissue is unclear, and I have a hard time seeing how this can be used to identify those at high and low AD risk as stated in the discussion.

Response: We agree with the reviewer that the clinical applicability of biomarkers derived from brain tissue is limited. However, such biomarkers remain valuable for elucidating disease mechanisms rather than for direct clinical use. To avoid any potential confusion, we have removed the ROC analysis from this revision.

2. In Figure 3a, it is clear that the scores would have to differ between cases and controls, as it is computed strictly from DMP that were identified in these datasets. It isn't clear whether or not these scores would generalize to other datasets, or whether the observed difference would be so pronounced.

Response: We appreciate this insightful comment. To evaluate the generalizability of the DNAm scores derived from the DMPs identified in our study, we tested their associations with AD pathology in two independent datasets:

(1) The Banner Sun Health Research (BSHR) cohort (N=244 postmortem brain samples; 117 inferior frontal gyrus and 127 superior temporal gyrus), and

(2) An independent sample from ROSMAP consisting of 100 postmortem brain samples (50 DLPFC and 50 striatum). These samples were previously assayed using the EPIC v1 array and were not part of the current analysis, which profiled 1,250 DLPFC samples using the EPIC v2 array in our discovery EWAS.

Both DNA methylation (measured by the Infinium MethylationEPIC v1 array) and neuropathology data were available in these two new datasets.

We replicated the association between the DNAm score and pathological diagnosis of AD across both datasets and multiple brain regions (see figure below), demonstrating the robustness of our findings. These results have been added to the revised manuscript (Fig. 2; Results, pages 7 [line 146-153]; Methods, page 21 [line 476-481]).

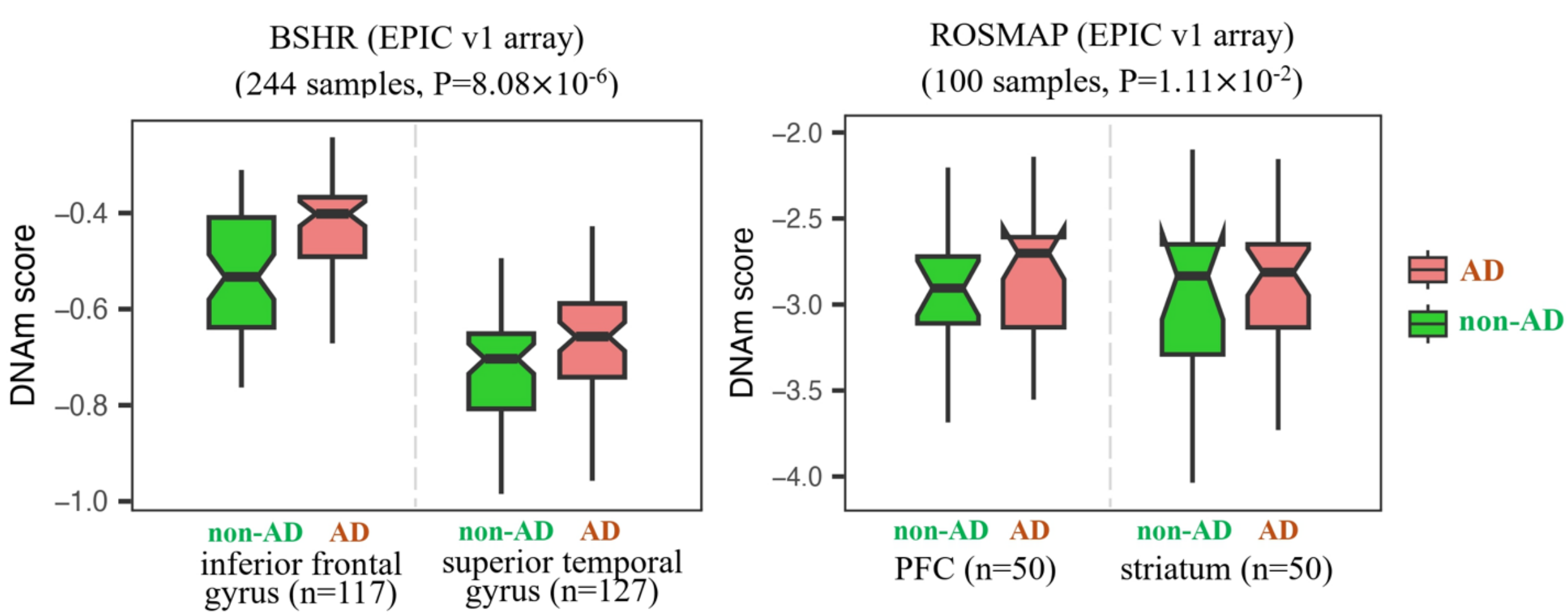

3. In the mediation analysis, the score is computed from AD-associated probes, and this score is tested as a mediator of the effect of the APOE genotype. This brings up the question as to whether the included DMP were APOE associated, AD associated, or both. Some examination of the APOE-associated vs. non-APOE-associated positions is warranted. It would also be worth commenting in the main text as whether or not any associated probes are in the APOE region itself (cis). From the supplement, the probes included in the score are for the most part not on chromosome 19. Do you expect so much trans regulation of DNA methylation based on prior studies of APOE and methylation?

Response: We truly appreciate this thoughtful comment. Among the 38 AD-associated DMPs identified in our EWAS, 23 (60.5%) were also significantly associated with *APOE ε4* (P-values ranged from 2.80×10^{-6} to 4.99×10^{-2}). The remaining 15 were associated with AD only.

All these 38 DMPs were not located on chromosome 19, where the *APOE* gene locates.

While the precise mechanisms are yet to be elucidated, several pathways (see the table below) may interpret the *trans* effect of DNA methylation on how *APOE* influences AD. This information has been added to the Discussion of the revised manuscript (page 11-12 [line 248-255]).

**Possible mechanism**	**How *trans* DNAm could affect *APOE*'s effect on AD**
Pathway modulation	Alter expression of genes in *APOE*-related pathways, e.g., lipid metabolism, inflammation, immune response, synaptic function, amyloid processing
Gene-gene interaction	*APOE* may interact with other AD risk genes (e.g., *BIN1*, *CR1*, *PICALM*), and trans DNAm may affect the expression or splicing of these interacting genes, thereby modifying the penetrance or strength of *APOE*'s effects.
Systemic or environmental influences	Trans DNAm may capture environmental exposures (e.g., smoking, diet, infection, stress) or systemic inflammation, could amplify or buffer *APOE*'s effects on AD.
Epigenetic moderation	Trans DNAm could partially mediate the relationship between *APOE* genotype and AD phenotype.<br>The level of trans methylation could also modify the strength of *APOE*'s effect.

4. The EPIC V2 array has multiple probes assessing the same position. Was this accounted for in the EWAS and DMR analysis?

Response: Thank you for this helpful comment. In our initial submission, we included all probes in the EWAS analysis and reported the most significant CpGs associated with AD pathology. In this revision, to address your concern, we excluded duplicate probes and retained only unique CpGs based on quality control metrics (detection *p*-values and bead counts). The Methods (page 17-18 [line 390-392]) and Results have been updated accordingly.

5. In several places, the paper states that DNAm mediates the APOE effect. It would be

better to state that it mediates a portion of the APOE effect throughout, to avoid giving the impression that methylation in the PFC explains the whole APOE effect on AD risk. It is worth noting in the discussion that when multiple brain regions are taken into account that the proportion of mediated variation could be higher.

Response: We appreciate this nice comment. As suggested, we have added "partially mediates" throughout the revised manuscript. We have also noted in the discussion that the proportion of mediated variation is likely to be larger when multiple brain regions are taken into account (page 13 [line 292-293]).

Reviewer #2 (Remarks to the Author):

This study presents a large-scale epigenome-wide association analysis of Alzheimer's disease (AD) neuropathology, integrating Illumina MethylationEPIC v2 arrays in a discovery cohort (n=1,250) with a smaller replication cohort (n=139), and further linking findings to single-nucleus RNA sequencing (snRNA-seq) data in a subset of the discovery cohort. The manuscript offers valuable insights into potential differentially methylated sites that might mediate known genetic risk (APOE). Overall, the study is timely and valuable to the neurodegenerative disease research community. However, several concerns and points need to be addressed.

Response: We appreciate the reviewer's positive comments on our manuscript.

Major comments:

1. In addition to DMP analysis, the authors should also perform DMR (differentially methylated regions) analysis, which reduces influence of single-site noise, and often align more with biological meaningful features.

Response: Thank you for the thoughtful suggestion. In response, we've included DMR analysis in this revision. The corresponding Methods (page 22 [line 489-494]) and Results (page 8 [line 163-169]) sections have been updated accordingly.

2. Please clarify the intended clinical utility of the methylation risk score and address the possibility of over-fitting. The 38 neuropathology-associated DMPs were discovered, replicated, and then reused to build risk models in the same two cohorts, an approach that can inflate performance estimates. In addition, an AUROC of ~0.72 for a model evaluated on its own training data appears low, and it is counter-intuitive that the AUROC is higher in the test set than in the training set.

Response: We appreciate this insightful comment. We agree that using DMPs identified within the same cohort could inflate the performance estimates of the risk model. In addition, in response to Reviewer #1's concern about the limited clinical utility of postmortem brain DNA methylation for risk prediction, we have removed the ROC analysis from the revised manuscript.

3. Because the prediction analyses were performed using brain samples, the translational value of the findings would increase if cross-tissue concordance and external performance in blood samples are assessed. Therefore, please quantify brain-to-blood correlations for the 38 replicated DMPs, construct a peripheral-blood methylation score using the CpGs that show robust concordance, and then evaluate that score in an independent blood DNA-methylation dataset (e.g., ADNI) to demonstrate its potential utility for risk stratification in living individuals.

Response: We are grateful for this valuable suggestion. In response to Reviewer #1's concern regarding the limited clinical utility of postmortem brain DNA methylation for risk prediction, as well as your concern about potential model overfitting, we have removed the risk model from this revision.

Meanwhile, we agree that it is important to examine the overlap between the 38 brain-derived DMPs and blood-based methylation data. To address this, we analyzed blood DNA methylation data from the ADNI cohort, testing the association between each individual CpG and incident AD diagnosis. Of the 38 AD-associated DMPs identified in our brain EWAS, blood DNAm levels of five CpGs (cg19803550, cg06362176, cg12476754, cg03499943, cg00521883) were nominally associated with incident AD ($P<0.05$). Among these, two CpGs (cg19803550, cg06362176) showed the same direction of association as in our brain EWAS, while three (cg12476754, cg03499943, cg00521883) showed the opposite direction. None remained significant after correction for multiple testing. These results are consistent with prior reports showing limited overlap of CpGs between brain and blood methylation in relation to AD, reflecting distinct DNAm profiles across tissues.[1,2]

Nevertheless, we constructed a blood DNAm score using the two CpGs with consistent directions of association (cg19803550, cg06362176) and found that this score was significantly associated with incident AD in ADNI (OR=1.57, P=0.004). These findings suggest that, while overlap between tissues is limited, some probes may hold promise for AD risk prediction and stratification.

4. Table 2 is currently missing. Please provide it and for each of the 38 replicated DMPs, add two annotations:

(1) Novelty status - Indicate whether the CpG has been reported in any previous brain EWAS of AD-related neuropathology.

(2) Array coverage - State explicitly whether the probe is present on the older Illumina 450 K array.
These columns will let readers see at a glance whether a CpG is "novel" because it was not significant in earlier 450 K analyses or because it was never assayed on the 450 K platform in the first place, and will highlight the findings most worthy of further follow-up.

Response: We apologize for the inadvertent omission of Table 2 in our initial submission, which occurred due to an administrative error during the file upload process. An updated Table 2 has been included in this revision.

5. In the mediation analysis, the authors computed "The DNAm score considers the association direction of 38 DMPs with AD neuropathology, which is defined as the sum of M-values of 10 DMP_pos minus Mvalues of 28 DMP_neg". Please explain why the methylation score was defined as the un-weighted sum of CpG M-values. Most methylation risk scores weight each CpG by its EWAS effect size (β-coefficient), see Huls et al., 2020 (PMID 31318318), to reflect the relative contribution of each probe. Please recompute the score using such weights or justify why an un-weighted approach is appropriate, and report whether the mediation results still holds.
Response: We are grateful for this suggestion. Following your recommendation, we have calculated a weighted DNAm score using the EWAS regression coefficients. The score remained significantly associated with pathological AD diagnosis, and the findings from our mediation analysis were also unchanged. These updates have been incorporated into the revised manuscript (Fig. 2, Results in page 7 [line 146-153]).

6. In addition, please consider performing single-CpG mediation tests to identify which of the 38 DMPs drive the aggregate effect.

Response: Following the reviewer's suggestion, we conducted single-CpG mediation analyses. Among the 23 DMPs associated with both *APOE* genotype and AD pathology, 20 were found to significantly mediate the relationship between *APOE* and AD diagnosis. These results have been added to the revised manuscript (page 8 [line 178-180]).

7. In the Methods section, the authors described two different meta-analytic frameworks: a random-effects model for the RADC + NBB analysis ("Meta-analysis") and a fixed-effects model for the three external DLPFC EWAS ("Comparison with previous EWAS results"). Please explain this discrepancy and consider applying the same model to both analyses.

Response: We appreciate this nice comment. In response to your comment #8, we have removed the fixed-effect meta-analysis of the three external DLPFC EWAS from this revision.

8. Also, please clarify the added value of the meta-analysis of the ROSMAP, London, and Mount Sinai DLPFC datasets when these cohorts have already been meta-analysed in prior publications. It's not clear why existing meta-analytic results were insufficient.

Response: We concur with this comment and have removed the fixed-effect meta-analysis of these three external DLPFC EWAS from this revision.

9. Please add a discussion comparing your findings with those of Shireby et al. 2022 (PMID 36153390), which mapped DMPs in purified brain cell types.

Response: We appreciate this insightful comment. Following your suggestion, we compared our results with those of Shireby et al. (2022), who conducted an EWAS of AD pathology using the Illumina EPIC v1 array in the Brains for Dementia Research (BDR) cohort.

Notably, Shireby et al. did not identify DMPs in purified brain cell types; instead, they used DNA methylation data from 12 DLPFC samples to estimate and adjust for cellular heterogeneity.

As shown in the figure below, 21 of the 38 DMPs (55.3%) identified in our study were also associated with AD pathology in the study by Shireby et al. ($P < 0.005$), exhibiting a consistent direction of effect, although some did not remain significant after correction for multiple testing.

We noted that differences in pathological assessments, participant characteristics, and covariates (e.g., cell type proportions, PMI) may contribute to the observed discrepancies. This point has been added to the revised manuscript (page 13 [line 280-284]).

Comparison between ROSMAP & NBB (our study; total 1,389 PFC brain samples) vs. BDR (Shireby, et al.; total 610 PFC brain samples)

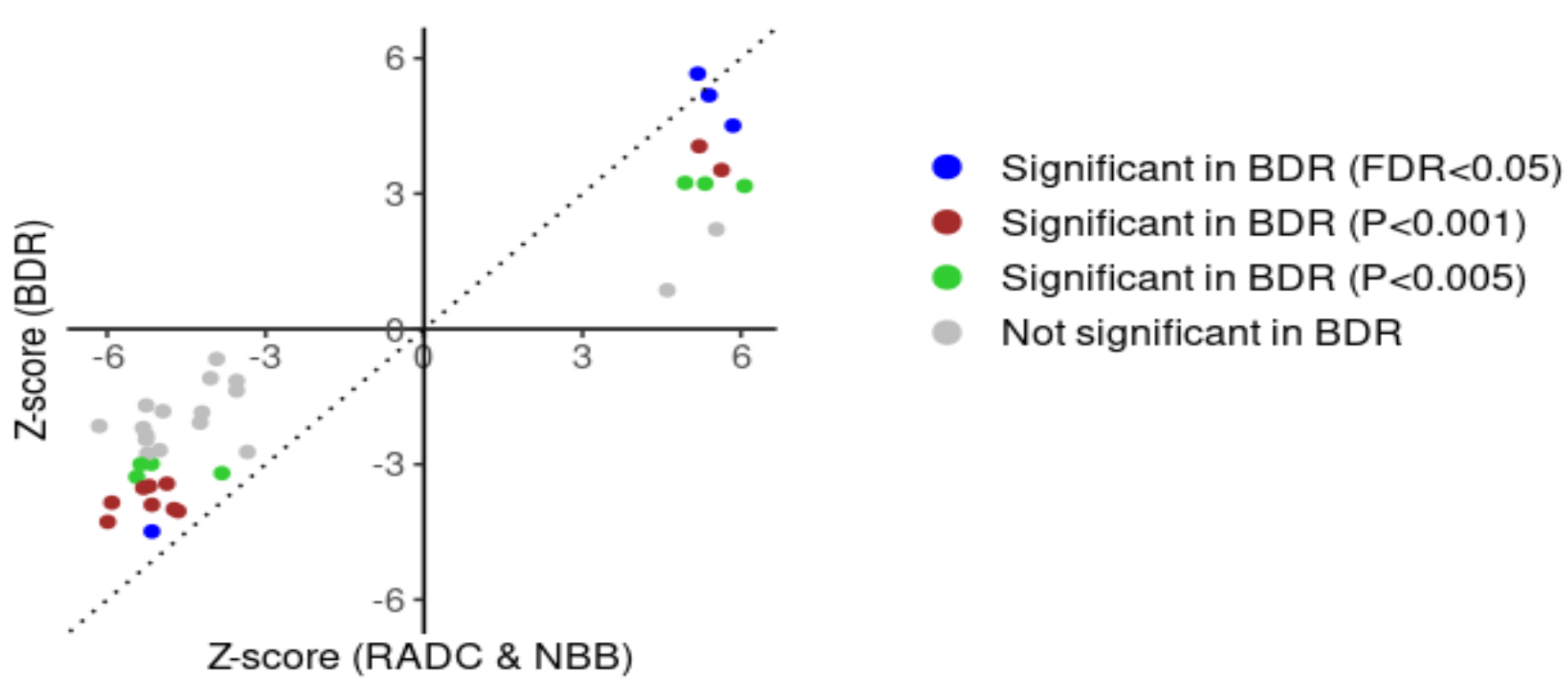

10. Please clarify why the enrichment was performed on GWAS variants that merely harbor the neuropathology-associated CpGs and why a nominal threshold of $P < 1 \times 10^{-4}$ was

chosen. A more biologically coherent strategy would be to base the test on (i) cis- or trans-mQTLs that regulate the significant CpGs, (ii) eQTLs or expression traits linked to those CpGs, or (iii) the CpGs themselves.

Response: We appreciate this insightful suggestion. In response, we have re-run the enrichment analysis using the following datasets:

(1) cis-mQTLs identified in the RADC brain samples and those reported in large-scale blood-based mQTL studies;

(2) cis-eQTLs from prior brain transcriptomic studies; and

(3) summary statistics from recent large-scale AD GWAS.

To assess the robustness of our findings, we also repeated the analyses using different significance thresholds. The updated methods and results are now presented in Supplementary Fig. 6 and Supplementary Table 10 (Methods, page 25 [line 560-568]).

11. Please comment on how the findings overlap across the three neuropathologic phenotypes. Discuss what this pattern of overlap, or lack thereof, suggests about common versus trait-specific molecular mechanisms.

Response: We appreciate this thoughtful comment. In the current study, we analyzed two quantitative measures of AD pathology (Aβ plaque burden, PHFtau tangle density) and pathological diagnosis of AD (yes/no) based on established neuropathological criteria.

Of the 38 DMPs identified in both RADC and NBB (total n=1,389), none was associated with all three neuropathologic phenotypes, six were associated with two pathologies, and 32 were associated with only a single pathology, despite the three phenotypes being significantly correlated (see figure below).

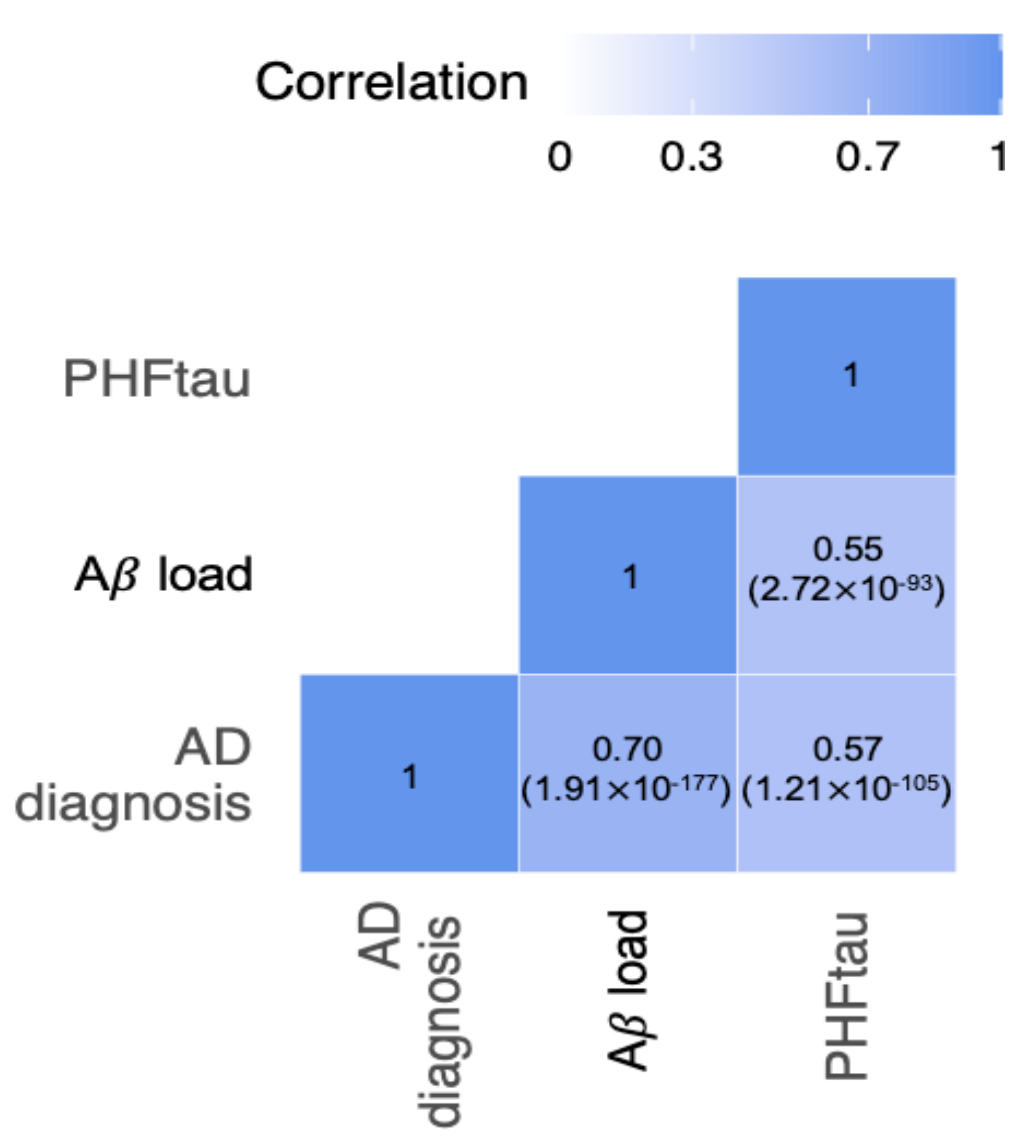

Figure. Heatmap showing the correlations between AD diagnosis, Aβ load, and PHFtau tangles in ROSMAP. Numbers in each box denote Spearman correlation (and P-value) between two neuropathological measures.

These results showed that a subset of CpG sites were significantly associated with more than one pathological measure, suggesting shared molecular mechanisms underlying multiple aspects of AD pathology. Of the 38 DMPs confirmed in both RADC and NBB samples, three of them (cg10045881, cg16215084, cg25917732) were associated with both Aβ and tau pathology, indicating common molecular pathways. These loci are annotated to immune- or inflammation-related genes (e.g., *CHI3L2*, *DDR1*), consistent with evidence that neuroinflammation contributes to both amyloid and tau pathology.

In contrast, we also identified CpGs that were uniquely associated with a single phenotype, suggesting trait-specific epigenetic alterations. For example, twenty out of 38 replicated CpGs were specifically associated with tau but not Aβ, potentially reflecting molecular mechanisms related to tau-specific neurodegeneration, such as intracellular microtubule dysfunction, prion-like propagation, and/or axonal transport. Similarly, fourteen CpGs were specifically associated with Aβ but not tau pathology, likely reflecting mechanisms linked to amyloid-specific pathology, such as APP processing, microglial sensing or clearance, and/or cerebral amyloid angiopathy. Notably, one CpG (cg00744233) was exclusively associated with overall pathological AD diagnosis, which may capture broader or cumulative pathological changes not fully represented by quantitative measures alone.

Taken together, the pattern of partial overlap among AD pathology-associated DMPs suggests that both shared and distinct epigenetic mechanisms contribute to the development of individual AD pathologies. These findings highlight the complex, multifactorial nature of AD and underscore the importance of examining multiple complementary phenotypes to gain a more comprehensive understanding of its molecular underpinnings.

This information has been added to the Discussion of the revised manuscript (page 12 [line 257-274]).

Minor comments:

1. Please supply the Supplementary Tables as Excel (.xlsx) files so that they are easier to read and reuse by other researchers.

Response: As suggested, we have provided the excel files for all supplementary Tables.

2. On line 70, add one sentence about how neuropathologic diagnosis of AD was defined.

Response: As suggested, we have added the definition of neuropathologic diagnosis of AD in both ROSMAP and NBB (page 5 [line 106-116]).

3. In Methods, it was mentioned “DNAm level at each probe was regressed against each AD neuropathologic measure using linear regression”. Please clarify was methylation beta values or M-values were used as outcome?

Response: We used M values as outcome in the model and have added this information to the revised manuscript (Methods in page 21 [line 465]).

4. In Table 1, tabulate race and ethnicity distribution of the subjects.

Response: We have added information for race and ethnicity to Table 1.

5. In legends of Figure 3, clarify which dataset was analyzed.

Response: We have added the information for datasets analyzed to the legend of Fig. 3.

6. In Figure 3, provide exact values for P-value.

Response: We have provided the exact value for *P*-value to Fig. 3.

7. On line 179, start a new paragraph.

Response: As suggested, we have started a new paragraph on line 275 in page 12 (previous line 179).

Reviewer #3 (Remarks to the Author):

Miao and colleagues present an interesting manuscript describing a DNA methylation, APOE genotype and AD neuropathology study on a large cohort of human postmortem brain tissue. The major novelties of this study are: 1) the use of the Illumina EPIC arrays V2, which have much higher coverage of methylation sites throughout the genome compared to the 450K arrays used in most of the previous large AD studies, and 2) the analysis considering the APOE genotype. Overall, this paper reports interesting findings, which can contribute to the advancement of knowledge regarding epigenetics of AD. However, I have identified several issues with room for improvement, which are listed below.

Response: We appreciate the reviewer’s positive comments on our manuscript.

1) The introduction is very brief (seems shorter than the abstract). It would be good to have a bit more context of previous findings from the largest AD studies, some of which looked not only at AD status, but also tau and amyloid pathology (e.g. Shireby et al 2022) and this should be made clear. Additionally, the authors should at least highlight DNA methylation changes in AD brain tissue studies that have been consistently reported across studies.

Response: In response to the suggestion, we have expanded the Introduction to include a summary of prior epigenetic studies in the brain and have incorporated the key publication reporting EWAS of various AD neuropathology measures (page 3-4).

2) In the results, it is not clear how the neuropathological diagnosis of AD was established and this is important for the interpretation of the findings and cross-study comparisons. This should therefore be made clear.

Response: As suggested, we have added the definition of neuropathologic diagnosis of AD in both ROSMAP and NBB (5-6 [line 106-116]).

3) Table 2 is missing and it is therefore not possible to assess the results that should be shown there.

Response: We apologize for the inadvertent omission of Table 2 in our initial submission due to an administrative error during the file upload process. In the resubmission, we have provided an updated Table 2.

4) In the general description of the EWAS findings (page 4), it is interesting to note the newly discovered associations. However, it would be good to see a bit more on description/discussion of the overlap with findings from previous AD EWAS and large meta-analyses. The 11 CpGs reported in eTable 4 and 7 CpGs in eFigure 4 seem quite a limited replication of previous findings, which is concerning. It is not even clear whether the direction of effect is the same as previously reported or not, which could mean even less overlap, and this should therefore be clarified. Wouldn't the authors expect more extensive overlap with previous studies (at least the larger ones)? It doesn't seem to be fully explained by the use of different arrays in the different studies as the new EPIC arrays are overall more comprehensive than the previous 450K not less. Please elaborate on the reasons for this very limited overlap.

Response: We appreciate this thoughtful comment. To address it, we've compared our results with previous AD EWAS including meta-analyses.[3,4]

Of the 109 DMPs identified in our meta-analysis (RADC+NBB), roughly half (57 DMPs) were also previously associated with AD pathology with a consistent direction of association. Some became non-significant after multiple testing correction.

Specifically, 36 (33%) of the 109 DMPs identified in our study (meta-analysis) were also significant in prior EWAS at either $P_{Bonferroni}$<0.05 or FDR<0.05. Notably, over 52% of the 109 DMPs were also associated with AD pathology in previous EWAS at $P<5\times10^{-4}$. The table below summarizes these comparisons. We've added this information to the Supplementary Table 6.

Table. Comparison of our results (ROSMAP+NBB, 109 DMPs identified in our meta-analysis) with previous EWAS (brain) on AD neuropathology

Previous EWAS [(ref)] [No. of brain samples]	Brain region	Significance thresholds in previous EWAS	No. of significant DMPs	Platform	No. (%) of DMPs overlapped with our study (109 DMPs)
Meta-analysis (2021)[3] [n=961]	PFC	$P_{Bonferroni}$<0.05	236	450K	11 (10.1%)
Meta-analysis (2021)[3] [n=1683]	PFC, temporal gyrus, entorhinal cortex, cerebellum	$P_{Bonferroni}$<0.05	220	450K	9 (8.3%)
Meta-analysis (2020)[4] [n=1030]	PFC	FDR<0.05	3,751	450K	24 (22%)
BDR EWAS (2022)[5] [n=610]	PFC	FDR<0.05	193	EPIC v1	7 (6.4%)
BDR EWAS (2022)[5] [n=1221]	PFC, occipital cortex	FDR<0.05	219	EPIC v1	10 (9.2%)
Meta-analysis with BDR (2022)[5] [n=2939]	PFC, occipital cortex, temporal gyrus, entorhinal cortex, cerebellum	FDR<0.05	2,253	450K	22 (20.2%)
**Overall overlap with previous DMPs after multiple testing**					**36 (33%)**
BDR EWAS (2022)[5] [n=610]	PFC	$P<5\times10^{-4}$	1,483	EPIC v1	25 (22.9%)
BDR EWAS (2022)[5] [n=1221]	PFC, occipital cortex	$P<5\times10^{-4}$	1,629	EPIC v1	23 (21.1%)
Meta-analysis with BDR (2022)[5] [n=2939]	PFC, occipital cortex, temporal gyrus, entorhinal cortex, cerebellum	$P<5\times10^{-4}$	2,736	450K	24 (22%)
**Overall overlap with previous CpGs significant at $P<5\times10^{-4}$**					**57 (52.3%)**

5) For the AD predictions using DNAm features (page 5), would it improve predictions if using all the CpG from the meta-analysis rather than the 38 DMPs found in both discovery

and replication cohorts? As the authors also looked at APOE status in this study, would the APOE status add anything to the AD predictions?

Response: Thank you for this thoughtful comment. In response to the concerns raised by Reviewers 1 and 2, we have removed the prediction model from this revision. We agree that incorporating additional CpGs or *APOE* genotype could potentially improve risk prediction, although the clinical utility of brain-derived biomarkers for this purpose remains limited.

6) Does the DNAm score composed of the 38 DMPs differ between APOE4 carriers and non-carriers?

Response: Thank you for your comment. DNAm score differs significantly between *APOE* $\varepsilon4$ carriers and non-carriers (P=6.36×10^{-7}). We have added this information to this revision (Fig. 3a).

7) Regarding the cell-type specific expression, eFigure 5 is incomplete (right end seems to be cut), and it should be explained what is represented in each of the three panels.

Response: We have double checked Supplementary Fig. 5 (previous eFigure 5) to ensure the figure is complete and added explanation to the figure.

8) In the genetic co-regulation and enrichment analysis (page 6), why was the GWAS threshold set to 1x10-4?

Response: Thank you for this helpful comment. In response to Reviewer 2's comment #10, we have updated the enrichment analysis. To examine the robustness of our findings, we have performed the enrichment analysis using different significance thresholds, and presented the results in Supplementary Fig. 6 and Supplementary Table 10 (Methods, page 25 [line 560-568]).

9) In the discussion (page 7, lines 144 to 149), there are several broad statements referring to "many genes", "several genes", "novel genes… many of which", it would be useful to have specific figures/numbers for each of these statements.

Response: We appreciate this suggestion and have added more precise information to the revised manuscript (page 10 [line 223-228]).

10) Also in the discussion (page 8, lines 175-6), given that this study investigated brain tissue, which is not easily accessible in living patients, I would suggest to tone down the

statement saying these findings "…may have potential value in risk stratification…", and recommend caution in interpretation without further investigation in accessible tissues.

Response: Thanks for the nice suggestion. We have revised the manuscript as suggested.

11) In the supplementary content, (Q1) in page 7, it is not clear how many tests were considered for the FDR correction, was it genome-wide for both discovery and replication cohort? (It is noted that for NBB, in eTable 2, unadjusted and FDR adjusted p-values are quite similar).

Response: Thank you for pointing this out. We have clarified the number of tests at each stage (discovery and replication) in the Methods (page 21 [line 473-475]) and added notes to Supplementary Tables 2-3.

11) (Q2) In page 9, why were cellular proportions (major contributors to variance in DNAm) not included in the regression models for the additional datasets?

Response: Thank you for pointing this out. All analyses in the current revision adjusted for estimated cell proportions.

11) (Q3) In page 11, it is not clear how mQTLs were generated.

Response: We appreciate this thoughtful comment. The mQTLs used in our analyses were derived from two sources: (1) mQTLs identified in an independent, unpublished study linking whole-genome sequencing and brain DNA methylation (DNAm) data generated from the RADC cohorts, and (2) mQTLs reported in previous large-scale mQTL studies in blood samples. This information has been included in the Methods section (page 23-24 [line 525-532], page 25 [line 560-568]).

11) (Q4) Also, it should be acknowledged that the datasets used for enrichment analyses (page 12), such as those used by GARFIELD, which are not brain related (eTable 9), and the Bellenguez et al. GWAS, which is not a pure AD GWAS (as it includes proxy cases, for example), have limitations and should be interpreted with caution.

Response: We appreciate this thoughtful comment. As Reviewer #2 also raised a question related to the enrichment analysis, we have re-run the enrichment analysis using the following datasets:

(1) cis-mQTLs identified in the RADC brain samples and those reported in large-scale blood-based mQTL studies;

(2) cis-eQTLs from prior brain transcriptomic studies; and

(3) summary statistics from recent large-scale AD GWAS.

To assess the robustness of our findings, we also repeated the analyses using different significance thresholds. The updated methods and results are now presented in Supplementary Fig. 6 and Supplementary Table 10 (Methods, page 25 [line 560-568]).

We note that GARFIELD annotates genetic variants (e.g., GWAS loci, eQTLs, mQTLs) and evaluates tissue- and cell type–specific enrichment patterns by integrating regulatory annotations from multiple tissues (e.g., brain, blood, liver) and cell types (e.g., immune cells, hepatocytes). Additional details on the GARFIELD framework and the datasets used have been added to the Methods section (page 25 [line 560-568]). We have also acknowledged the inclusion of non-brain data resources in GARFIELD as a study limitation in the Discussion (page 13 [line 294-297]).

References

1 C Silva, T. *et al.* Cross-tissue analysis of blood and brain epigenome-wide association studies in Alzheimer's disease. *Nat Commun* **13**, 4852 (2022). https://doi.org/10.1038/s41467-022-32475-x

2 Hannon, E., Lunnon, K., Schalkwyk, L. & Mill, J. Interindividual methylomic variation across blood, cortex, and cerebellum: implications for epigenetic studies of neurological and neuropsychiatric phenotypes. *Epigenetics* **10**, 1024-1032 (2015). https://doi.org/10.1080/15592294.2015.1100786

3 Smith, R. G. *et al.* A meta-analysis of epigenome-wide association studies in Alzheimer's disease highlights novel differentially methylated loci across cortex. *Nat Commun* **12**, 3517 (2021). https://doi.org/10.1038/s41467-021-23243-4

4 Zhang, L. *et al.* Epigenome-wide meta-analysis of DNA methylation differences in prefrontal cortex implicates the immune processes in Alzheimer's disease. *Nat Commun* **11**, 6114 (2020). https://doi.org/10.1038/s41467-020-19791-w

5 Shireby, G. *et al.* DNA methylation signatures of Alzheimer's disease neuropathology in the cortex are primarily driven by variation in non-neuronal cell-types. *Nat Commun* **13**, 5620 (2022). https://doi.org/10.1038/s41467-022-33394-7

Response to Reviewers' Comments

We thank the editors and reviewers for their thorough and constructive evaluation of our manuscript. We have carefully addressed all comments and suggestions, and provide a detailed point-by-point response below. The revised manuscript is submitted alongside this response, with all changes highlighted in blue for ease of review. Unless otherwise stated, all page and line numbers, and the order of tables and figures, refer to the revised manuscript. Authors' responses are indicated in blue.

Reviewer #4 (Remarks to the Author):

1. In the methods the preprocessing is described as being QN followed by BMIQ using wateRmelon. WateRmelon has a number of QN based methods, the authors should say which one they did use.

Response: We thank the reviewer for this helpful clarification request. We used the **"dasen"** method within the *wateRmelon* R package for quantile normalization. We have revised the Methods section to state this explicitly (page 18, line 404).

2. From github I can see the answer to 1 is Dasen. The code availability is exemplary. The authors should include a download to the repo in supplementary materials to give easy, guaranteed access to the code.

Response: We thank the reviewer for this constructive suggestion and for taking the time to examine our code repository. Following this recommendation, we have archived the complete preprocessing and analysis code to ensure permanent, guaranteed access. Specifically, we created a tagged release of our GitHub repository and deposited a frozen snapshot in Zenodo, which has assigned a permanent DOI (https://doi.org/10.5281/zenodo.20558402). A direct link to this archive has been added to the **Code Availability** statement (page 26, lines 591–593).

Reviewer #5 (Remarks to the Author):

1. Reviewer 2 - comment 11 - amendments to manuscript on page 12. The authors have added to the discussion in the revised manuscript, reflecting on the CpGs uniquely associated with different phenotypes. The authors comment that one CpG (cg00744233) was exclusively associated with overall pathological AD diagnosis [so not with amyloid or tau], and also about others that are associated with more than one trait. However, in reality, what they are referring to here is possibly reflecting this CpG meeting the Bonferroni

significance threshold in only this analysis [but it may be showing pathology-associated changes just missing significance in the other regions]. The authors should explore for each of the 38 DMPs whether they were showing a similar pattern in the other phenotypes (ie is the direction of effect the same as expected, and whether these were at least nominally significant) to be able to claim CpGs are exclusively unique. Effect size and p value for the other comparisons should be added to the relevant supplementary tables for the analysis of each phenotype, and this reflected on in the discussion, if seen.

Response: We thank the reviewer for this important clarification. We agree that our original use of "exclusively/uniquely associated" was imprecise — these CpGs reached Bonferroni-corrected significance in only one of the three neuropathological analyses, but may still show consistent, if sub-threshold, associations with the other phenotypes.

To address this, we examined all 38 replicated DMPs across all three phenotypes (neuropathologic AD diagnosis, Aβ load, and PHFtau tangles), evaluating direction of effect, FDR significance, and nominal significance. As shown in the new **Supplementary Fig. 4**, effect directions were concordant across all three phenotypes for all 38 DMPs. Moreover, 36 of 38 DMPs (94.7%) reached nominal significance ($P < 0.05$) across all three phenotypes, though some did not survive multiple testing correction (Supplementary Fig. 4). Effect sizes (regression coefficients) and P-values for all DMPs across all three phenotype analyses have been added to **Supplementary Table 4**. The Results (pages 6-7, lines 139–145) and Discussion (page 12, lines 263–276) have been revised accordingly to emphasize the directional consistency of these associations.

2. Reviewer 3 - comment 1 (changes page to manuscript in pages 3-4). The authors have added a nice background about other studies in this area. There is one slightly misleading statement. In line 62 the authors say "CpG sites within several gene cluster, such as ANK1, RHBDF2 and HOX". However, the previous EWAS in field have not seen changes in ANK1 or RHBDF2 gene clusters, just in these genes. In addition, brain EWAS have highlighted the HOXA cluster (whilst the HOXB has been reported in blood. The authors should rephrase this to say "CpG sites within several genes, such as ANK1, RHBDF2 and the HOXA gene cluster".

Response: We thank the reviewer for catching this imprecision. We have revised the sentence (page 3, line 62) to read: *"CpG sites within several genes, such as ANK1, RHBDF2 and the HOXA gene cluster"*, which more accurately reflects the scope of prior brain EWAS findings.

3. Reviewer 3 - comment 11 - changes to manuscript on page 9 line 202-205. The authors add details about the results of this analysis, however these are vague and simply state they saw significant enrichments. Can they state here in the results what these were.

Response: We thank the reviewer for this helpful suggestion. We have expanded the Results section (page 9, lines 193–200) to explicitly state the significant enrichments identified, rather than characterizing them in general terms. Briefly, we found that, "after correction for multiple testing, significant enrichments were observed across multiple GWAS significance thresholds, predominantly in active regulatory regions, including accessible chromatin (peaks, hotspots, and footprints), transcription start site (TSS) and enhancer chromatin states, as well as activation-associated histone marks (H3K4me3, H3K27ac, and H3K9ac), indicating that these DMPs preferentially localize to regions with active transcriptional regulatory function".

4. Amendments to page 13 - lines 280-284. The addition of this section has made the next sentence confusing "Future research should incorporate single-cell technologies and examine additional brain regions, as in a recent study, despite its smaller sample size" Can the authors clarify what they are referring to by "despite its smaller sample size".

Response: We thank the reviewer for flagging this ambiguity. The phrase "despite its smaller sample size" was intended to acknowledge that a recent single-nucleus multi-omic study (Liu Z et al., *Cell*, 2025; 188:4980–5002.e29), which profiled snRNA-seq and ATAC-seq data across six brain regions in 111 AD and control individuals, demonstrated the value of multi-region epigenomic profiling at single-cell resolution, albeit at a smaller scale than bulk-tissue EWAS such as the current study (n=1,250). We recognize, however, that framing this as a sample size comparison was misleading: the more limited sample size in that study is an expected consequence of the substantial cost and technical complexity of single-nucleus sequencing, not a reflection of its scientific merit. We have removed the phrase "despite its smaller sample size" and revised the sentence accordingly (page 13, lines 293–296).

5. I have one point relating to the meta-analyse of the two cohorts (RADC and NBB). Generally meta-analyses in just two cohorts are avoided, particularly if one study is a lot larger (which is the case here) as it can essentially influence the pooled effect size, which will be driven by the larger cohort. What was the rationale for meta-analysis, rather than simply replication?

Response: We thank the reviewer for this methodological observation. We agree that a meta-analysis across only two cohorts of substantially different sizes is problematic, as the pooled effect estimate would be heavily driven by the larger cohort (RADC). We have therefore removed the meta-analysis and instead treat NBB as an independent replication

cohort, which more appropriately reflects the study design and the relative contributions of each cohort. The manuscript has been revised accordingly.

6. I have another query about how adjusted for race in the EWAS - it looks like the linear models simply used a binary variable (white vs non-white). Was there a reason why the authors did not use genetic PCs to account for ethnicity?

Response: We thank the reviewer for this important methodological query. Among the 1,250 RADC participants included in our EWAS, 1,166 (93.3%) self-identified as non-Hispanic whites, 66 (5.3%) as African Americans, 5 (0.4%) as American Indians or Alaska Natives, and 13 (1%) as other racial groups. Genetic data (WGS) was unavailable for 323 participants (26%). Given this missing data and the predominantly White composition of the cohort, we adjusted for self-reported race as a binary variable (White vs. non-White) as an alternative.

To assess whether this choice materially affected our results, we conducted a sensitivity analysis restricted to RADC participants with available genetic data (n=927), replacing the binary race variable with the first five genetic PCs (Model 2) while keeping all other covariates identical to the primary model (Model 1). As shown in the scatterplots below (Fig. 1, Supplementary Fig. 8 in the revised manuscript), effect sizes were highly concordant between the two models across all three phenotypes (AD diagnosis, Aβ load, and PHFtau tangles): points clustered tightly around the line of identity, the direction of association was fully concordant, and nearly all CpGs remained nominally significant (*P* < 0.05) under both adjustment strategies. These results indicate that the binary race covariate provides adequate control for population stratification in this cohort and that our primary findings are not sensitive to the choice of ancestry adjustment method. We have added these results from sensitivity analyses to the Results section (pages 9-10, lines 201–210).

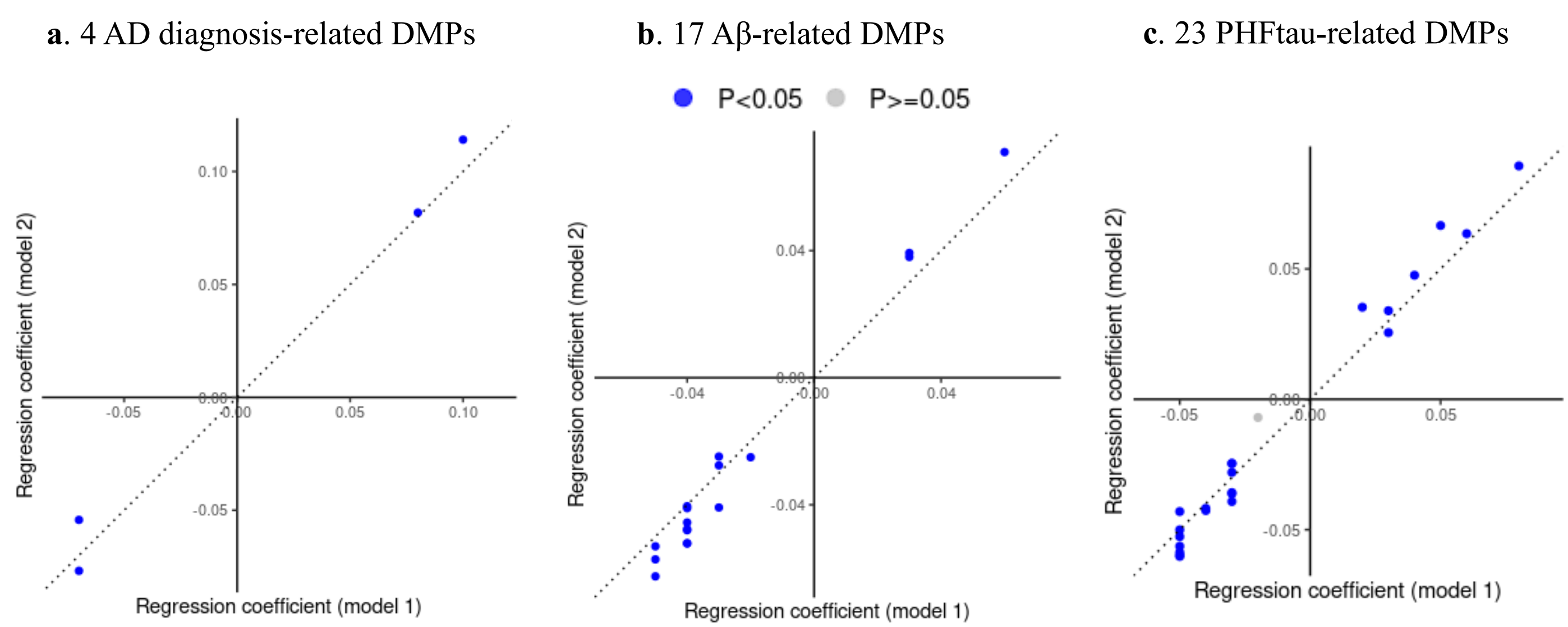

Fig 1. Concordance of DMP effect sizes between models adjusting for binary race (White vs. non-White) and adjusting for the first five genetic PCs. Scatterplots compare regression coefficients from the primary model adjusting for self-reported race as a binary variable (White vs. non-White; Model 1, x-axis) against those from a sensitivity model adjusting for the first five genetic principal components (PCs; Model 2, y-axis). Each point represents a replicated DMP reaching significance in both discovery and replication cohorts under Model 1. The dotted line denotes the line of identity (y = x). Points are colored by significance in Model 2: blue indicates $p < 0.05$ and grey indicates $p \geq 0.05$. Across all three phenotypes (AD diagnosis, Aβ load, and PHFtau tangles), effect sizes were highly concordant between the two models, with 100% agreement in the direction of association, demonstrating that our primary findings are robust to the choice of ancestry adjustment.
